# Supplementary material for: A programmable chemical computer with memory and pattern recognition
Source: Nat Commun. 2020 Mar 18;11:1442. doi: 10.1038/s41467-020-15190-3 (PMC7080730; doi:10.1038/s41467-020-15190-3)
Supplement: Supplementary file 1 — Supplementary Information [file 41467_2020_15190_MOESM1_ESM.pdf]

Supplementary Information for

"A Programmable Chemical Computer with Memory and Pattern Recognition"

Juan M. Parrilla,[1] Abhishek Sharma,[1] Soichiro Tsuda,[1] Geoffrey J. T. Cooper,[1] Gerardo Aragon-Camarasa,[1] Kevin Donker1 and Leroy Cronin[1]*

[1] *School of Chemistry, University of Glasgow, Glasgow, G12 8QQ, UK*
**Corresponding author e-mail: lee.cronin@glasgow.ac.uk*

Contents

Supplementary Methods 3
1 Overview of the automated platform 3
1.1 Description of the physical parts required to build the platform 4
1.2 Electronics 14
1.3 Software 15
1.4 Bill of materials 18
2 Image processing 19
2.1 Camera and configuration used 19
2.2 Detection of oscillations 20
2.3 Generation of time-maps 21
3 Chemistry 24
3.1 Preparation of the reagent solutions for the BZ reaction 24
3.2 Experimental protocol 24
4 Experimental data 25
4.1 Studying the relation between arena architecture and oscillations 25
4.2 Input patterns used for pattern recognition 27
4.3 Studying the colour changes based on the input recipe 28
4.4 Reaction-diffusion experiments in a device without walls 35
4.5 Testing for how long the BZ system can oscillate 35
4.6 Studying the memory effect of the BZ medium 35
4.7 Studying the speed of stir rotation and its relation to BZ oscillations 42
4.8 Studying the stability in the frequency of the BZ oscillations 48
4.9 Programming a pattern sequence into the BZ medium 49
4.10 Experimenting with water medium instead of BZ medium 52
5 Data analysis 53
5.3 Using CNN to train the BZ medium 54
5.4 Using an Autoencoder to digitally generate BZ oscillations 54

5.5 Mutual information calculations ... 56
6 Towards generalized computation using the BZ platform ... 56
6.1 Experimental inputs ... 56
6.2 Chemical states ... 57
6.3 Mathematical description of the dynamics of coupled BZ Oscillators ... 58
7 Estimating number of operations and required logic gates ... 61
8 Power dissipation in BZ reaction ... 63
Supplementary Note 1: Supplementary Figure enhancements ... 64
Supplementary References ... 65

Supplementary Methods

1 Overview of the automated platform

This section explains the different parts of the automated platform in detail. This platform is the one responsible for performing the physical experiments, and programming the inputs of the chemical computer processor which is housed in the reaction ‘arena’ containing the array of stirrer bars. These stirrer bars are housed in the fluidically interconnected grid. This section will cover the three main domains that make up the platform:

- The physical parts, which are a mixture of 3D-printed parts and hardware parts which are commercially available plus a set of DC motors and magnetic stirrers.
- The electronics, which are based on the Arduino microcontroller plus a custom-made PCB.
- The software to run the electronics which includes the firmware to run the Arduino board, and the Python script which drives the DC motors. This can be programmed so that complex input patterns can be used.

The fully integrated system also needs the chemical reaction details, artificial intelligence algorithms, and computer vision software to read out the outputs of the chemical computations. These are explained in later in sections 2, 3 and 5. This section will focus on how to build the automated platform. Supplementary Figure 1 shows the three domains that will be covered in this section. The physical parts which are where the experiments are done, the electronics which actuate the DC motors, and the software, which is responsible for communication between the Arduino and the motors, as well as between the computer and the Arduino motor-programmer.

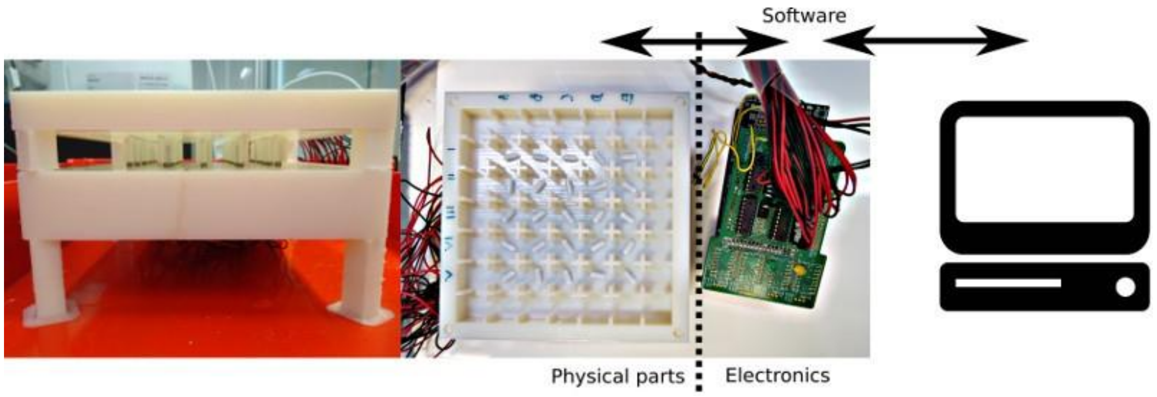

Supplementary Figure 1: Outline of the platform showing the three main elements needed to build the physical parts, the electronics and the software.

1.1 Description of the physical parts required to build the platform

The construction of the physical setup is explained in this section. This involves producing 3D printed parts, and the DC motors and stirrer bars, as well as explaining how the DC motors are connected to the Arduino board.

3D printed parts

All the following 3D-printed parts were designed using the OpenScad software and printed with either a Stratasys Connex500 3D printer using the VeroWhitePlus material or using a RepRap i3 3D-printer, and using transparent polylactic acid (PLA). Supplementary Figure 2 shows a photo of the final platform, showing all the different 3D-printed parts that will be discussed in this section: the stirrer bar array, spacers, motor magnet mount, motor holder array and stand or 'legs' that support the chemical processor or reaction 'arena'. The 3D printer files are available from the authors on request but screen shots of the Supplementary Source Code to produce the files are given here.

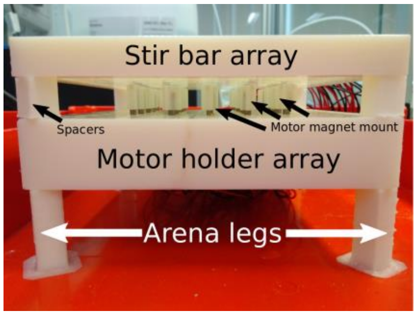

Supplementary Figure 2: Photograph of the assembled platform, highlighting the different 3D printed parts that will be discussed in this section.

The main two 3D printed parts are the DC motor holder array, where the DC motors are inserted, and the stirrer bar array, which lays on top of the other one, and contains the chemical reaction arena. Supplementary Figure 3 shows the design of the piece that will hold the motors. It contains an array of 7 by 7 cells, although only the central 5 by 5 array in the middle is used. The DC motors were inserted through the square cells, and their shaft would show through the holes. These squares and holes were designed to match the specification of the motors used (see Section 1.1).

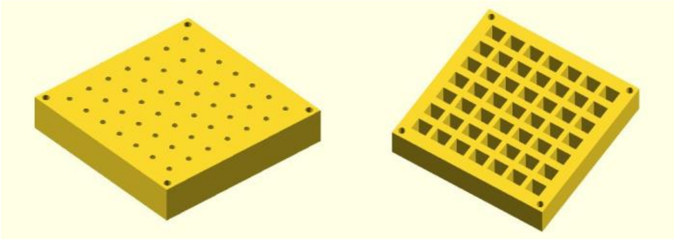

Supplementary Figure 3: 3D design of the piece that holds the motors.

The following OpenScad Supplementary Source Code (Supplementary Source Code 1) shows all the variable definitions used in OpenScad in order to generate the motor holder matching our DC motors, as well as the stir bar array which will be discussed next. In this code, all the numbers given represent distances or sizes in millimetres. The following OpenScad Supplementary Source Code (Supplementary Source Code 2) can generate the designs shown in Supplementary Figure 3 exactly.

The 3D designs shown in Supplementary Figure 3 were 3D printed as described, and the results can be seen in Supplementary Figure 4. The left part of this Supplementary Figure shows the bottom of the piece, where the motors were inserted. The right part of this Supplementary Figure shows the top of this piece, where it can be seen how the motor's shafts go through the 3D printed holes. The other 3D printed parts that can be seen in this Supplementary Figure will be discussed in this section.

Supplementary Source Code 1: 3D designs specifications

```
//Cell dimensions
nx = 7;     //number of cells in x
ny = 7;     //number of cells in y
cx = 16;     //cell width
cy = 16;     //cell length
cz = 11;     //cell depth
//Arena total size is (nx*cx)x(ny*cy)
//Cells are (cx-0.5gw)x(cy-0.5gw)
//Base and edge thicknesses
base = 1.5;
edge = 15;
//Equidistant groove depth and width
gw = 2;     //width
gz = 0;     //depth
gh = 0;     //height above cell edge
shim = 0.0;     //narrowing wrt grooves
extend = 0;   //extension of grooves into sides
raise = -2;      //height of channels from bottom of cell [mm]
prop = 0.5;     //propoprtion of cell wall to have open as channel
//Stirrer motor parameters
md = 4;     //motor top sleeve diameter
mx = 12.05; //motor x width
my = 10.05; //motor y width
mz = 24;    //motor length
//Bolt hole paramters
boltd = 5.2;  //bolt diameter
bolte = 5;  //distance from edge
//Calculate arena dimensions
ax = nx*cx;
ay = ny*cy;
az = cz + gz;
//Calculate block dimensions
bd = sqrt((ax*ax+ay*ay))+edge;   //diameter
bz = az + base;     //depth
```

Supplementary Source Code 2: Motor Holder OpenScad code

```
difference(){
  //Create block
  translate([-edge/2,-edge/2,bz*5])
  cube([ax+edge,ay+edge,mz],false);
  //Subtract bolt holes
  translate([-edge/2+bolte,-edge/2+bolte,bz*5-1])
  cylinder(h=mz+2,d=boltd,$fn=50);
  translate([ax+edge/2-bolte,-edge/2+bolte,bz*5-1])
  cylinder(h=mz+2,d=boltd,$fn=50);
  translate([-edge/2+bolte,ay+edge/2-bolte,bz*5-1])
  cylinder(h=mz+2,d=boltd,$fn=50);
  translate([ax+edge/2-bolte,ay+edge/2-bolte,bz*5-1])
  cylinder(h=mz+2,d=boltd,$fn=50);
  //Subtract motor holes
  for (i=[1:nx]){
    for (j=[1:ny]){
      translate([(i-0.5)*cx,(j-0.5)*cy,bz*6-1])
      cube([mx,my,mz],true);
    }
  }
  for (i=[1:nx]){
    for (j=[1:ny]){
      translate([(i-0.5)*cx,(j-0.5)*cy,bz*5-1])
      cylinder(h=mz+2,d=md,$fn=50);
    }
  }
}
```

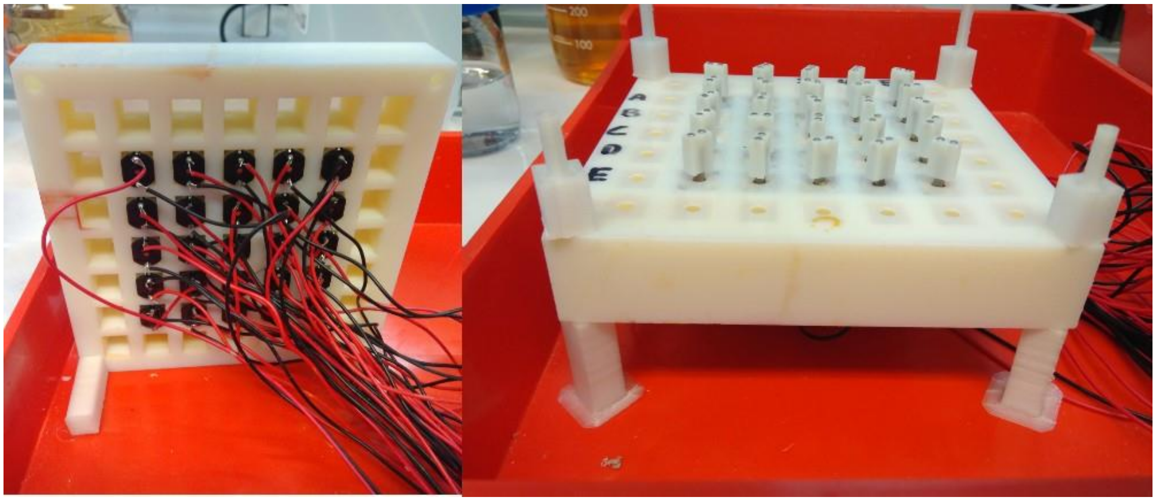

Supplementary Figure 4: Photograph of the 3D printed motor holder. Left: The bottom of the piece, where the DC motors were inserted. Right: The top part of the piece, where the shafts coming through the holes can be seen.

To describe the stirrer bar array, we will first need to show the empty arena, which can be seen in Supplementary Figure 5. The specifications of this design can be seen in the Supplementary Source Code 1. This device was not used for the chemical computations described in the manuscript, but it was required

for us to develop the final system. This is because some preliminary reaction-diffusion experiments were tested to develop the system, see Section 4.4.

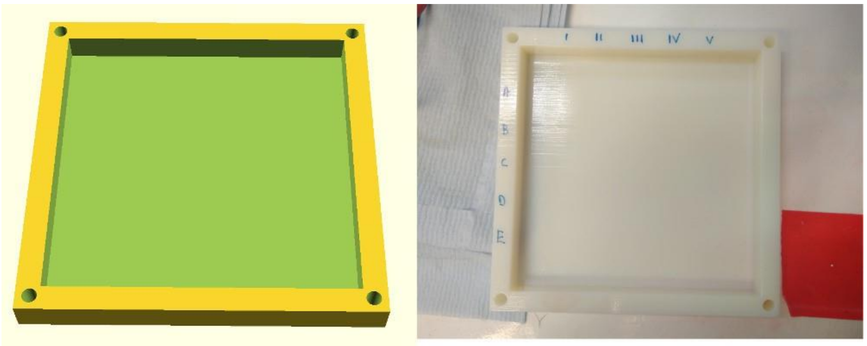

Supplementary Figure 5: The 3D design of the stirrer bar array, in this case, only the empty arena is shown. The left image shows the 3D design, while the right image shows the 3D printed piece.

Supplementary Source Code 3 details how to design this piece using OpenScad. The variables shown here were defined previously in Supplementary Source Code 1. Supplementary Figure 6 shows the 3D designs of the main stirrer bar arrays used during the experiments. As before, only the 5 by 5 cells in the middle were used. In these cells, a stirrer bar was placed as described in Supplementary Figure 6. This Supplementary Figure shows the two main designs that we used in this research. In the left one, the gap between the cells is completely open, and the fluid is free to move throughout the array. In the right one, the gaps between the cells were narrowed restricting fluid flow. The specification of this design in terms of distances and sizes was described in the Supplementary Source Code 1.

Supplementary Source Code 4 details how to design a device like the one shown in the left part of Supplementary Figure 6. This code uses the variables defined in Supplementary Source Code 1 and the empty arena detailed in Supplementary Source Code 3. To close the gap between cells, as shown for example on the right in Supplementary Figure 6, the variable named “raise” in Supplementary Source Code 1 was increased. Supplementary Figure 7 shows the 3D printed device as described or shown on the left in Supplementary Figure 6. This is the main device that was used to do the chemical computations described in the manuscript. Supplementary Figure 8 shows other devices that were designed and 3D-printed, but have not been used during the research described in the main manuscript, but instead used to validate the approach. These are shown to show how easy it is to create different architectures, and hence create customised versions of the arena.

Supplementary Figure 9 shows the 3D design of the magnet holders that were inserted into the shafts of the motors. The two holes in the top contained magnetic bars, while the bottom hole is the one where the motor shaft was inserted. The magnetic bars used were sourced from “First4Magnets”. They are described as “2mm dia x 2mm thick N42SH Neodymium Magnet - 0.15kg Pull”. Supplementary Source Code 5 details

how the magnet holders that were attached to the shafts of the motors was designed. As before, all the distances and sizes are represented in millimetres.

The last two pieces to detail are the “arena legs” which are responsible of holding up the whole chemical processor platform, and align the motor holder part, and the stirrer bar array as well as the 3D-printed “spacers”. These spaces are needed to create enough space between the motor holder part and the stirrer bar array part so that the shafts of the motors, with their magnetic holders, can be attached and can freely rotate. These need to be as close as possible to the stirrer bars placed in the stirrer bar array part, so that the rotation of the shafts will translate to efficient rotation of the stirrer bars above them. Supplementary Figure 11 shows the 3D designs of these pieces. Supplementary Source Code 6 shows the OpenScad code use to design these pieces, as well as their dimensions. Supplementary Figure 12 shows the 3D-printed pieces. The “arena legs” and the “spacers” parts were 3D-printed using a RepRap i3 3D-printer, and using transparent polylactic acid (PLA). Once all the pieces are 3D-printed, it is very straightforward to assemble the final structure.

Supplementary Source Code 3: Empty arena OpenScad code

```
difference() {
  //Create block
  union() {
    translate([-edge/2,-edge/2,0])
    cube([ax+edge,ay+edge,bz],false);
  }
  //Subtract arena
  translate([(0.5*gw),(0.5*gw),base])
  cube([(ax-gw),(ay-gw),(az+1)],false);
  //Subtract bolt holes
  translate([-edge/2+bolte,-edge/2+bolte,-1])
  cylinder(h=bz+2,d=boltd,$fn=50);
  translate([ax+edge/2-bolte,-edge/2+bolte,-1])
  cylinder(h=bz+2,d=boltd,$fn=50);
  translate([-edge/2+bolte,ay+edge/2-bolte,-1])
  cylinder(h=bz+2,d=boltd,$fn=50);
  translate([ax+edge/2-bolte,ay+edge/2-bolte,-1])
  cylinder(h=bz+2,d=boltd,$fn=50);
}
```

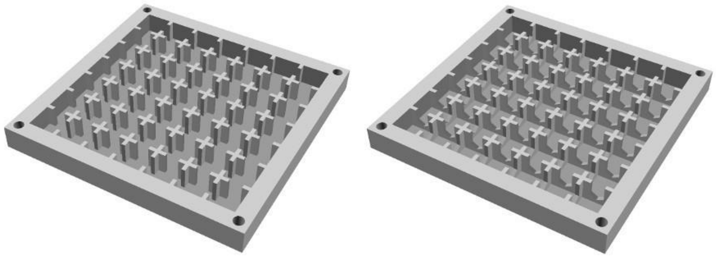

Supplementary Figure 6: The 3D design of the stir bar array. These are the two main designs shown in the manuscript. In the left one, the gaps between the cells are completely open, and the fluid can go through. In the right one, the gaps have a barrier, limiting the fluid flow between the cells.

Supplementary Source Code 4: Stir bar array OpenScad code

```
//Walls and Channels
difference() {
  //Walls
  union() {
    //Build X grid walls
    if (nx>1) {
      translate([cx-(gw*0.5)+shim,-(extend-shim),(base)])
      cube([gw-(2*shim),ay+((extend-shim)*2),(az+gh-(gz*2))]);
      for (i=[2:(nx-1)]) {
        if (i<nx) {
          translate([(i*cx)-(gw*0.5)+(shim),-(extend-shim),(base)])
          cube([gw-(2*shim),ay+((extend-shim)*2),(az+gh-(gz*2))]);
        }
      }
    };
    //Build Y grid walls
    if (ny>1) {
      translate([-(extend-shim),cy-(gw*0.5)+shim,(base)])
      cube([ax+((extend-shim)*2),gw-(2*shim),(az+gh-(gz*2))]);
      for (i=[2:(ny-1)]) {
        if (i<ny) {
          translate([-(extend-shim),(i*cy)-(gw*0.5)+(shim),(base)])
          cube([ax+((extend-shim)*2),gw-(2*shim),(az+gh-(gz*2))]);
        }
      }
    };
  }
  //Channels
  union() {
    //Cut X grid wall channels
    if (nx>1) {
      //subtracts a vertical stack of 4 hexagonal prisms half a diameter apart
      for (m=[1:(ceil(1/prop)*2)]){
        //channels (gaps) in x as single long triangular prisms
        for (l=[1:nx]){
          translate([(l-0.5)*cx,0,(m*prop*cx*0.5)+gz+base+raise-3])
          rotate([-90,90,0])
          cylinder(h=ay,d=(prop*cy),$fn=6);
        }
      }
    }
    if (ny>1) {
      //subtracts a vertical stack of 4 hexagonal prisms half a diameter apart
      for (m=[1:(ceil(1/prop)*2)]){
        //channels (gaps) in x as single long triangular prisms
        for (l=[1:ny]){
          translate([0,(l-0.5)*cy,(m*prop*cy*0.5)+gz+base+raise])
          rotate([0,90,0])
          cylinder(h=ax,d=(prop*cx),$fn=6);
        }
      }
    }
  }
}
```

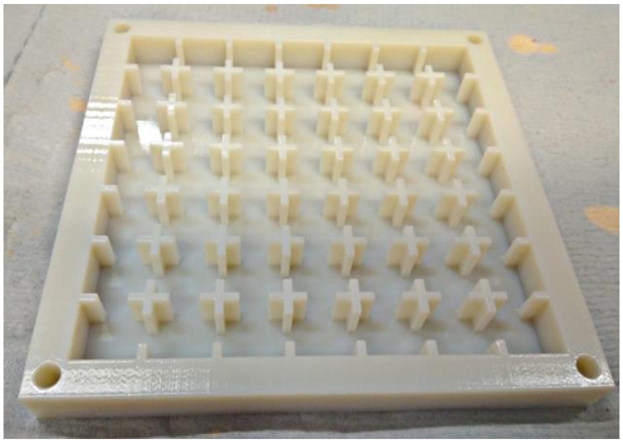

Supplementary Figure 7: The 3D printed device as described in Supplementary Source Code 4 and show as a schematic in Supplementary Figure 6.

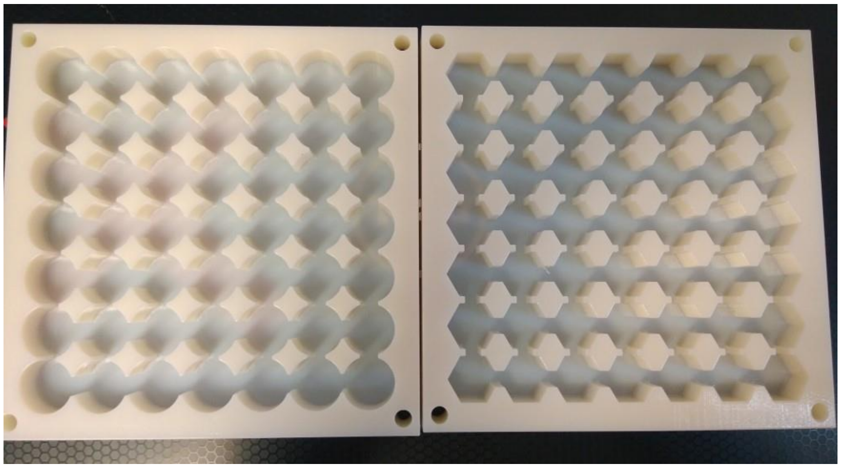

Supplementary Figure 8: Examples of other devices that were designed and fabricated, but that were part of the research explained in the main manuscript.

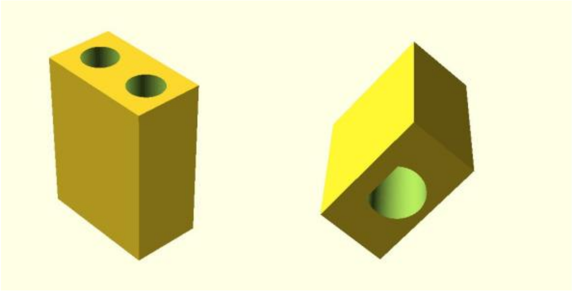

Supplementary Figure 9: The 3D design of the magnet holder that is attached to the motor shaft. The two holes in the top contained small magnet bars, and the bottom hole was inserted into the motor shaft.

Supplementary Source Code 5: Magnet holder OpenScad code

```
//Motor shaft paramters
shtd=3;       //shaft diameter
shtl=7;       //shaft length

//Magnet parameters
magd = 2.06;       //magnet diameter (cylinder)
magh = 2;       //magnet height (cylinder)
magx = 1;       //magnet width (cube)
magy = 1;       //magnet length (cube)
magz = 1;       //magnet depth (cube)
mags = 1.5;       //magnet spacing

//Holder parameters
hldd = 7;           //holder diameter (cylinder)
hldh = 2.5;           //holder height (cylinder)
hldx = 4;           //holder width (cube)
hldy = 7;           //holder length (cube)
hldz = 10;           //holder depth (cube)

//Construct
difference(){
  //Holder
  translate([hldd/2,hldd/2,0])
  cylinder(h=hldh,d=hldd,center=false,$fn=150);
  //Magnets
  translate([x/2,y/2-magd/2-mags/2,z-magh+0.1])
  cylinder(h=magh,d=magd,center=false,$fn=50);
  translate([x/2,y/2+magd/2+mags/2,z-magh+0.1])
  cylinder(h=magh,d=magd,center=false,$fn=50);
  //Shaft
  translate([x/2,y/2,-0.1])
  cylinder(h=shtl,d=shtd,center=false,$fn=150);
}
//shaft catch
cube([(hldx-shtd)/2+0.55,hldy,shtl],center=false);
```

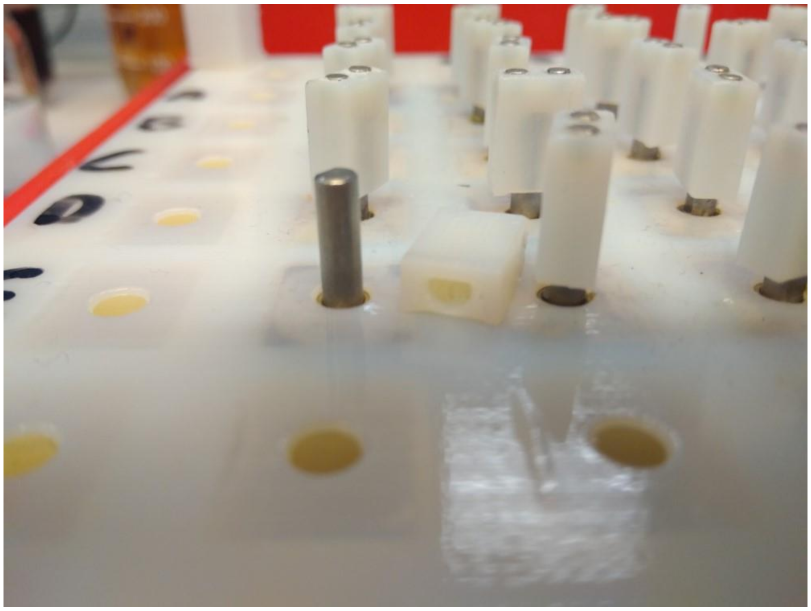

Supplementary Figure 10: The 3D printed device as shown in Supplementary Figure 9. In this Supplementary Figure it can be seen how the final design looked, and how it was attached to the motors.

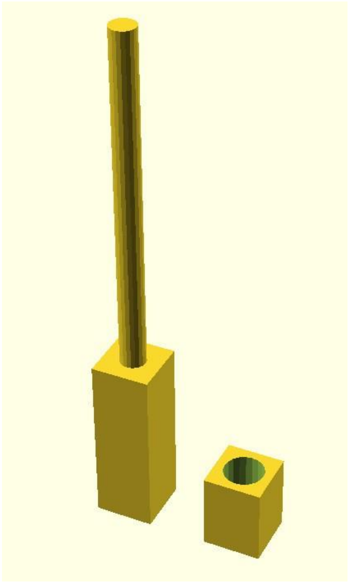

Supplementary Figure 11: The 3D designs of the arena legs (left) and spacer (right).

Supplementary Source Code 6: Arena legs and spacers OpenScad code

```
// Arena legs
cube([10,10,30]);
translate([5,5,30])cylinder(h=60, d=4.5, $fn=20);
// Spacers
difference() {
  cube([10,10,13.5]);
  translate([5,5,-1])cylinder(h=20, d=7, $fn=20);
}
```

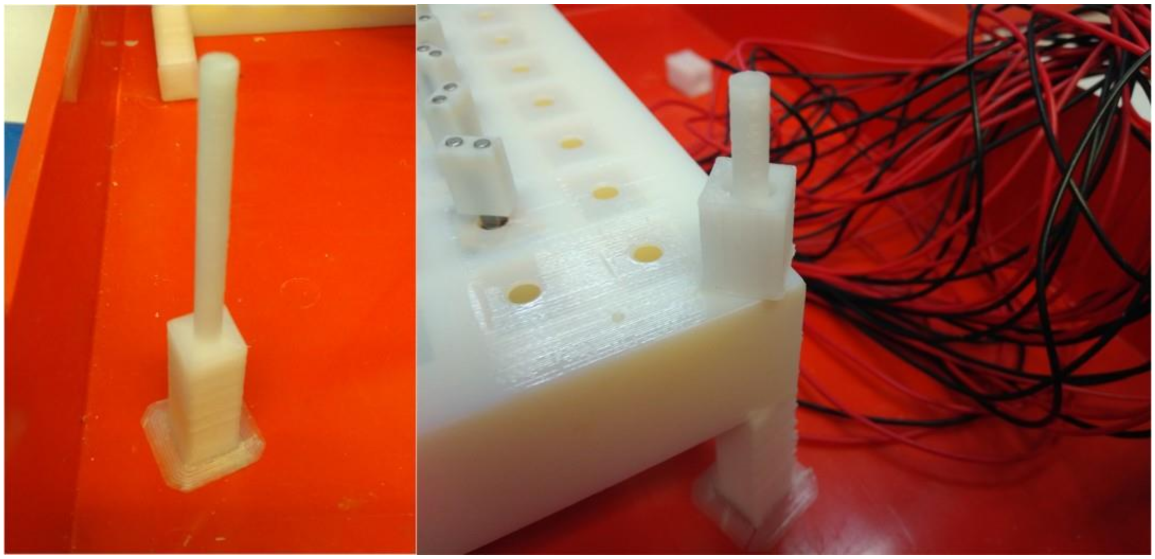

Supplementary Figure 12: The 3D printed pieces of the arena legs. Legs on the (left) and spacer (right).

In summary, the overall platform shown in Supplementary Figure 2 has:

- 1x Stir bar array
- 1x Motor holder array
- 4x Arena legs
- 4x Spacers
- 25x Motor magnet mount
- 50x Magnet bars

DC motors

The DC motors used were bought from “AliExpress” under the following description: “DC 6V 200RPM Mini Metal Gear Motor With Gearwheel Model:N20 3mm Shaft Diameter”. Supplementary Figure 13 shows the motor specifications from the seller’s website. All the 3D printed pieces which used the motors (motors holder array and magnet mounts) were designed based on these specifications. Supplementary Figure 14 shows a photo of the actual motors. These motors were inserted into the motor holder array as can be seen in Supplementary Figure 4, and the magnet mounts were attached to their shafts as can be seen in Supplementary Figure 10. As their specifications show, when supplied with 6V they are expected to rotate at 200 RPM. We visually calibrated them to rotate at 90 RPM by modulating the PWM signal generated by the electronics (see Section 1.2). This motor speed was chosen because it generated sSupplementary Table BZ oscillations in the array (see Section 4.7) that could be easily tracked visually.

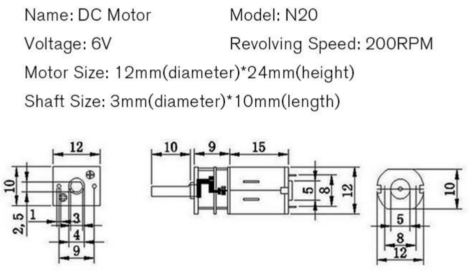

Supplementary Figure 13: DC motor specifications from the seller’s website.

Stirrer bars

The stirrer bars used in our platform were sourced from “Fisherbrand”. They are described as “magnetic follower, micro” with a size of 8 by 3 mm. Our objective was to get the largest possible stirrer bars so that the quantity of fluid transferred would be maximized, while at the same time being small enough so that their magnetic fields don’t interfere with each other. Supplementary Figure 15 shows these stirrer bars placed in the “stirrer bar array”.

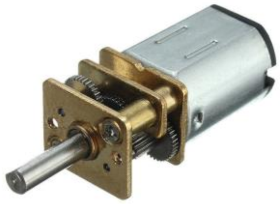

Supplementary Figure 14: Photo of one of the DC motors used in this platform.

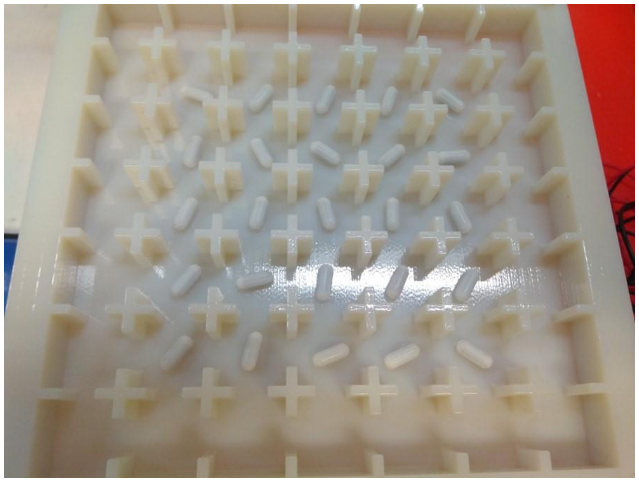

Supplementary Figure 15: Photo showing the stirrer bars placed in the “stirrer bar array”.

1.2 Electronics

In order to turn the motors, we based the electronics on the Arduino microprocessor boards. In this platform we used the Arduino Mega board. To achieve precise motor control we opted to use pulse width modulation (PWM) signals that can be generated in an Arduino in order to control the voltage received by the motors. As shown in Section 1.1, when these motors receive 6 volts they rotate at 200 RPM, but they can be rotated at lower speeds by supplying a lower voltage. Because we needed to actuate 25 motors, and this is more than an Arduino can control via its default pins, we used the Arduino compatible shield “Adafruit 16-Channel 12-bit PWM/Servo Shield - I2C interface”. This shield expands Arduino’s functionality to 16 different PWM signals, which is still not enough, but these shields can be connected and stacked to increase the number of PWM signals available. In our case, we used two shields in total, generating a total of 32 different PWM signals, which exceeds the 25 we need for the platform. Finally, in order to transform these PWM signals into voltages, we designed a shield (Supplementary Figure 16). This shield used four “ULN2803A Darlington Transistor Arrays”, which were used to transform the PWM signals into voltages. Supplementary Figure 17 outlines our set-up. Supplementary Figure 18 shows a picture of the actual electronics. The connection between our purpose designed PCB and the DC motors was achieved via a wiring loom see Supplementary Figure 4.

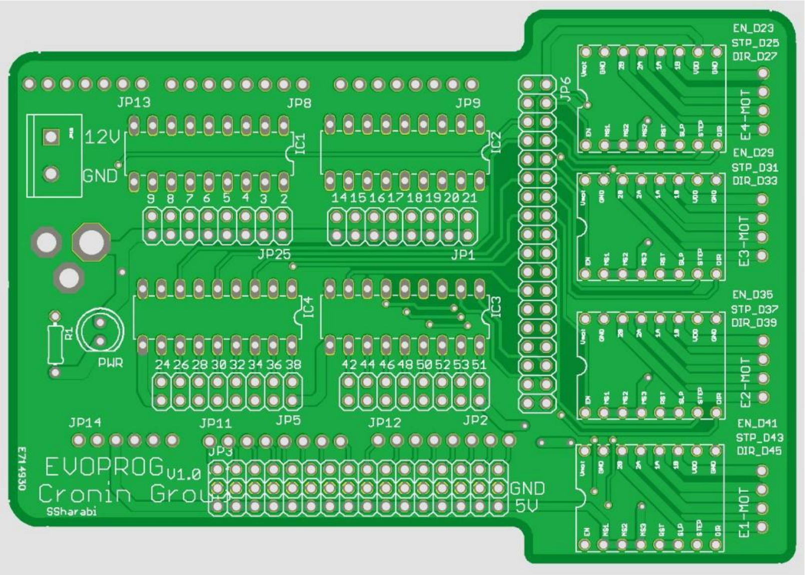

Supplementary Figure 16: The PCB shield designed by us to transform the PWM signals into voltages using ULN2803A Darlington transistor arrays. Further schematics to aid reproduction of this are available from the authors on request.

The power supply used in this platform was a "80W Switched Mode DC Multi Voltage Slim Bench Power Supply". In particular, the model "N27GG". It was set to "6.09V", and the range was set to "16V 5A".

1.3 Software

The software layer responsible for controlling the described platform can be divided into two parts:

- The firmware that runs in the Arduino board. It is responsible for generating the electronic pulses that actuate the motors.
- A Python script that runs on a computer connected through USB to the Arduino board. This Python script aims to provide a more user-friendly level abstraction to the actuation of the platform.

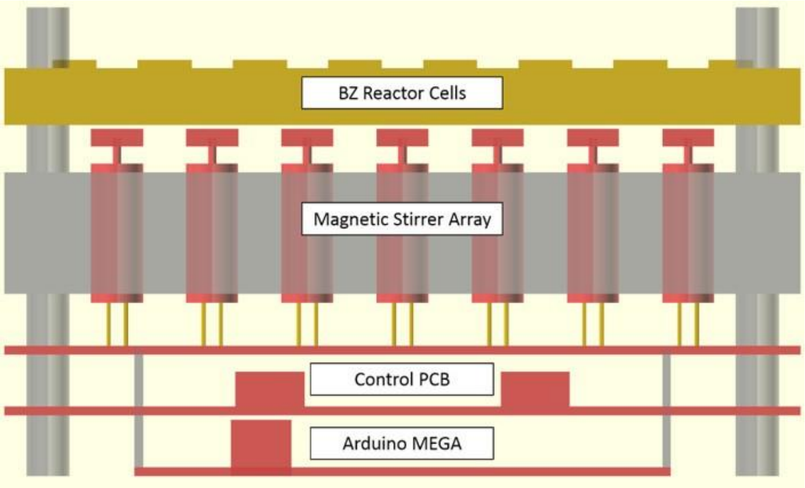

Supplementary Figure 17: Outline of the electronics setup used.

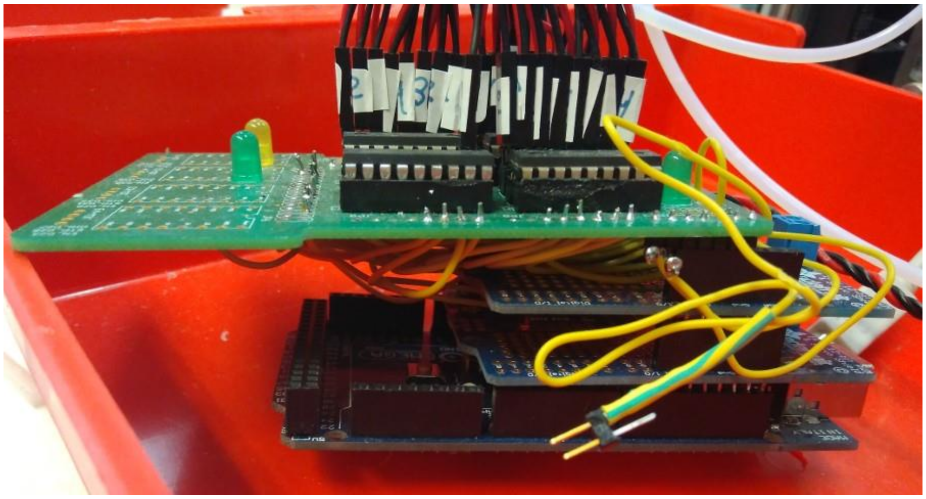

Supplementary Figure 18: Picture of the electronic system used, which is based on the Arduino platform. From top to bottom: Arduino Mega, Adafruit 6-Channel 12-bit PWM/Servo Shields, Adafruit 6-Channel 12-bit PWM/Servo Shields and our purpose designed PCB.

Firmware

The main objective of the Firmware is to generate the different signals that will actuate the motors and it is based on the standard Arduino syntax and libraries. Also, because we are offering a higherlevel interface in Python to the user, the firmware is also responsible for reading the commands generated by the user, decode them, and generate the relevant electronic signals. In this way the chemical computer can be programmed at a high level away from the Arduino using a purpose built system.

As described during the Electronics section (Section 1.2), in order to generate PWM signals that would actuate the motors, we used an "Adafruit 16-Channel 12-bit PWM/Servo Shield - I2C interface". Thus, in order to send commands to this PWM Shield, we used its corresponding Arduino library:

https://github.com/adafruit/Adafruit-PWM-Servo-Driver-Library. With this library loaded, in order to send different commands for PWM signals, we will need to use the function “setPWM(pin, 0, speed)” where pin refers to the particular motor we want to actuate, “0” should always be zero, and speed is the factor that will generate the PWM signal. In our case, speeds lower than 500 would not be able to actuate all motors, and the maximum available value is 4000. Because our platform is using two of this Adafruit boards, our firmware contains two Adafruit objects, which we called “pwm0” and “pwm1”.

To get all the motors to rotate at a similar speed, we aimed for all of them to require a PMW signal of around 500, and this translates to around 90 revolutions per minute. Because all the motors are slightly different, the actual value was calibrated individually, on a motor by motor basis. The speed of 90 revolutions per minute was the standard speed used throughout all the experimentation, except when described otherwise and hence can be considered to be our standard base speed.

Finally, the firmware is also responsible for decoding messages from the user which would specify which motor to actuate and at which speed. The syntax of these commands is similar to GCode and it is detailed in Supplementary Table 1. In the case of the pin number, Adafruit shield 0 accepts values from 0 to 15, while Adafruit shield 1 accepts values from 0 to 8, because there are 25 motors in total. In the case of the PWM signal, the Adafruit library accepts any value between 0 and 4000, but our experience with the particular DC motors used is that any value lower than 500 might not achieve actuation.

Code	Description
AX	Selects Adafruit shield X. $X \in [0, 1]$
PY	Selects pin Y. $Y \in [0, 15]$
SZ	Selects PWM signal. $Z \in [0, 4000]$

Supplementary Table 1: “AX PY SZ” Example of G-code operations decoded by the firmware layer.

Following this syntax, the Arduino board would receive a command like “A1 P8 600” through the USB serial connection. Then, the firmware decodes this command and generates the corresponding PWM signals using the Adafruit software library as explained. In this case, for example, it will use the software object “pwm1” (because the parameter “A” is given a “1”), and then execute the command “pwm1.setPWM(8, 0, 600)”.

Python user interface

The objective of this layer is to offer to the user a higher-level interface in order to actuate the platform. It offers an API to the user with commands like “actuate motor” or “disable motor”, and then transforms these commands into a G-Code command as explained in the previous section. Supplementary Table 2 describes the API of the Python user interface used to control the platform. This user interface will either be used by a user, or by any other higher-level decision-making layer.

A user would for example execute the function “activate motor(”A1”, 600)”. The first thing this Python interface does is to translate from ”A1” to Adafruit board and pin number, which in this case refers to board number 1, and pin number 8. Therefore, a command like “A1 P8 600” is sent to the Arduino through the USB serial connection.

Code	Description
init__(port)	Connects to the serial port “port” and loads a dictionary which relates from motor codes such as “A1” or “E5” to its corresponding Adafruit shield and pin
del__	Disables all the motors and closes the serial connection
close()	Same as del__
Activate_motor(code, speed)	Activates motor “code” at speed “speed”
Disable_motor(code)	Disables motor “code”
Activate_all(speed)	Activate all the motors at speed “speed”
Disable_all()	Disable all the motors
Pattern_from_file(file)	Loads the JSON “file” which should specify a pattern. Returns a pattern object
Activate_pattern(pattern, speed)	Activates the motors following the pattern “pattern” at the speed “speed”

Supplementary Table 2: bzboard.py API.

1.4 Bill of materials

- 25x Fisherbrand magnetic follower, micro. 8 by 3 mm.
- 25x DC 6V 200RPM Mini Metal Gear Motor With Gearwheel Model:N20 3mm Shaft Diameter.
- 1x Motor array (3D-printed)
- 1x Stir bar array (3D-printed)
- 4x Arena legs (3D-printed)
- 4x Legs spacers (3D-printed)
- 25x Motor shaft magnet holder (3D-printed)
- 50x First4Magnets 2mm dia x 2mm thick N42SH Neodymium Magnet - 0.15kg Pull.
- 1x Arduino Mega 2560
- 2x Adafruit 16-Channel 12-bit PWM/Servo Shield - I2C interface.

- 1x PCB shield designed by ourselves which was used to transform the PWM signals into voltages. It used ULN2803A Darlington Transistor Arrays.
- 1x Computer running Python 3 and OpenCV 3.
- 1x Microsoft LifeCam Cinema Web camera.

2 Image processing

The image processing software layer is responsible for:

- Detecting the BZ oscillations with image processing. In the case of this project, the objective was to assign the state of the cells as being either "red" or "blue".
- Using the oscillation data from a whole experiment, we generated a "time-map" plot, which is a very common visualization technique in BZ experiments where the oscillations are plotted against time.

2.1 Camera and configuration used

A Microsoft LifeCam Cinema Web camera was situated 12.5 cm above the arena in order to record the experiment. While the experiment proceeded, the video stream from the camera was fed into a computer running a Python (3.6.5) OpenCV (3.4.1) script, which generated a video file for the experiment. In the majority of the experiments the video was recorded first, and then the oscillation detection and data analysis were performed later, but it was possible to operate the system in real time for closed loop experiments. The video was conSupplementary Figured to 800 by 600 pixels, and 30 frames per second (FPS) and XVID compression was used. Supplementary Figure 19 shows the positioning of the camera with respect to the platform 12.5 cm above the centroid of the arena. The camera was conSupplementary Figured using the software Guvcview and Supplementary Figure 20 shows the configuration parameters used.

A Microsoft LifeCam Cinema Web camera was situated 12.5 cm above the arena in order to record the experiment. While the experiment happened, the camera stream was fed into a running Python (3.6.5) OpenCV (3.4.1) script, which generated a video file of the experiment. In the majority of the experiments the video was recorded first, and then the oscillation detection and data analysis were performed later. The video was conSupplementary Figured to 800 by 600 pixels, and 30 frames per second (FPS). XVID compression was used. Supplementary Figure 19 shows the positioning of the camera respect to the platform.

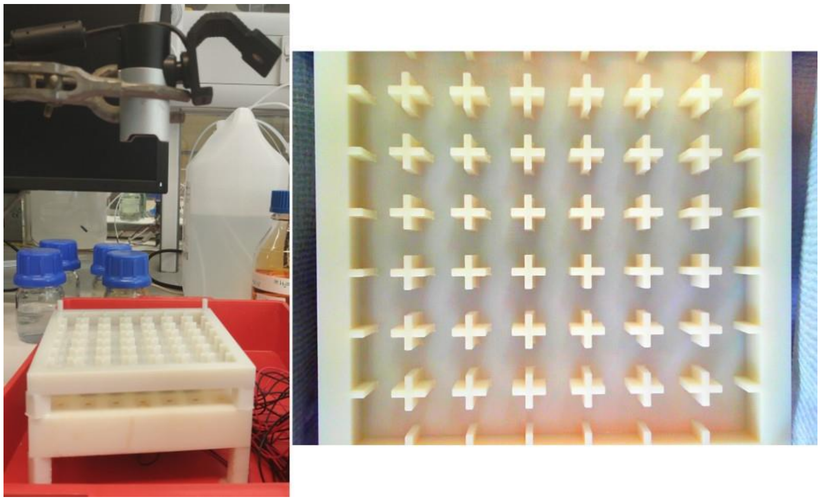

Supplementary Figure 19: Left: Picture of the platform and positioning of the camera, which is 12.5 cm above the arena. Right: Picture of the arena as seen from the camera.

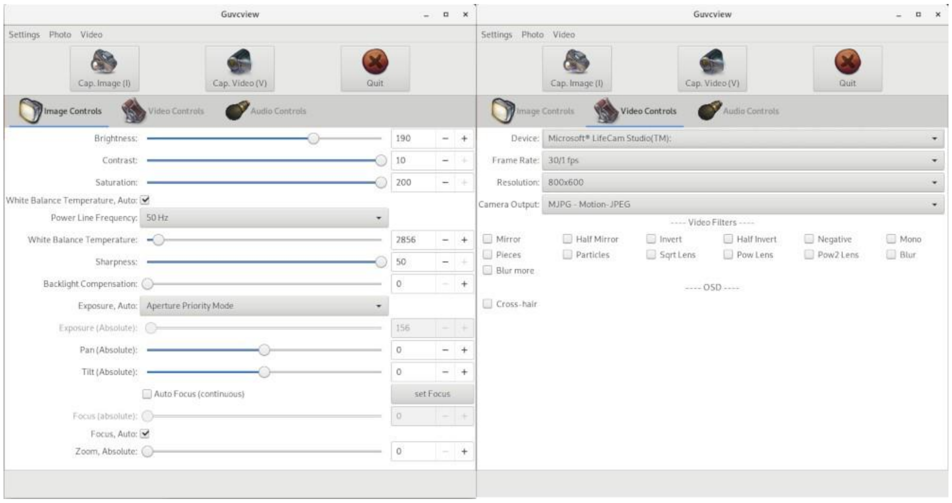

Supplementary Figure 20: Camera configuration using the software Guvcview.

2.2 Detection of oscillations

This section focuses on how computer vision algorithms were used in order to detect the BZ oscillations. These BZ oscillations were labelled as red or blue. The objective of this software layer is to, given a camera frame like the one shown in Supplementary Figure 21 – left, output the classification of the cells between red and blue, as shown in Supplementary Figure 21 – right.

Initially, the problem of segmenting between red and blue might look simple, but if we look at Supplementary Figure 22, for example, we can see that very often just based on the colour it is difficult to say if a cell is blue or red. The cause of this is that the BZ reaction colour changes over time (see Section 4.3). This change depend on many factors, among them, the number of motors enabled, and their respective speeds. It would be possible to model all these variables and obtain for any given configuration its expected colour, but in this type of scenarios, it is often easier to use machine learning. In this case, we used a support vector machines

(SVM) to help us assign / classify the states of the cells. The reason for choosing an SVM is because they are able to classify such states, they are very well integrated within the OpenCV library, they work very small for small datasets, and once trained they can be used extremely quickly. This last point is important because in some of our experiments we will need to classify the cells at a rate of 30 FPS.

In order to train the SVM a dataset was built. This dataset was built by a human researcher who labelled different cells, in different videos, at different points of the BZ reaction. In total, 1541 samples of "red" were labelled, and 888 of "blues". These samples looked like the square cells shown in Supplementary Figure 22. In order to provide more information to help the training step, the average colour of the whole BZ medium during the previous 3000 frames for each of the cells was also recorded and used. In particular, the blue and red channel average values were stored as part of the file name, with values between 0 and 255.

In order to train the SVM, firstly the samples as shown on Supplementary Figure 22 were equalised using CLAHE (as implemented in OpenCV). The clip limit was set to 1, and the tile Grid Size was set to 4 by 4. The next step consisted of transforming the RGB data from an image into the input data that could be used with the SVM. Several different transformations were tested, such as only taking the blue channels, or a PCA, but ultimately we obtained the best results using a 3D histogram in the HSV colour scheme; 8 bins were used in the histogram for each of the channels. This histogram was then input to the SVM, with the average colour of the previous 3000 frames in the red and blue channel, as explained before.

The SVM library used was the one included in OpenCV. An RBF kernel was used, and the type was set to "C SVC". The C parameter was set to 5, and the gamma parameter was also set to 5. An exploration was done to find these two parameters, and these two values were the ones that resulted in a better model. The results could be seen in Supplementary Figure 21. Although here only images are shown, the code was designed to work specifically with the video data.

2.3 Generation of time-maps

The generation of time-maps is a very common and powerful way of representing the behaviour of the BZ reaction oscillations as a function of time. The objective is to take a given video and generate a plot that shows how the system oscillates through time, see Supplementary Figure 23.

In order to achieve the generation of the time maps for a given video, an algorithm was used that would take five columns of pixels for every frame. Each of these columns was centered around one of the columns of the 5 by 5 arena. Then these five columns would be stitched together in a single column. Then the algorithm would take the next frame, and repeat the process. The new columns generated would be placed next to the previous one, see Supplementary Figure 24. This algorithm can be applied to raw videos, like the one shown in Supplementary Figure 23, or to already segmented videos, like the example shown in Supplementary Figure 25.

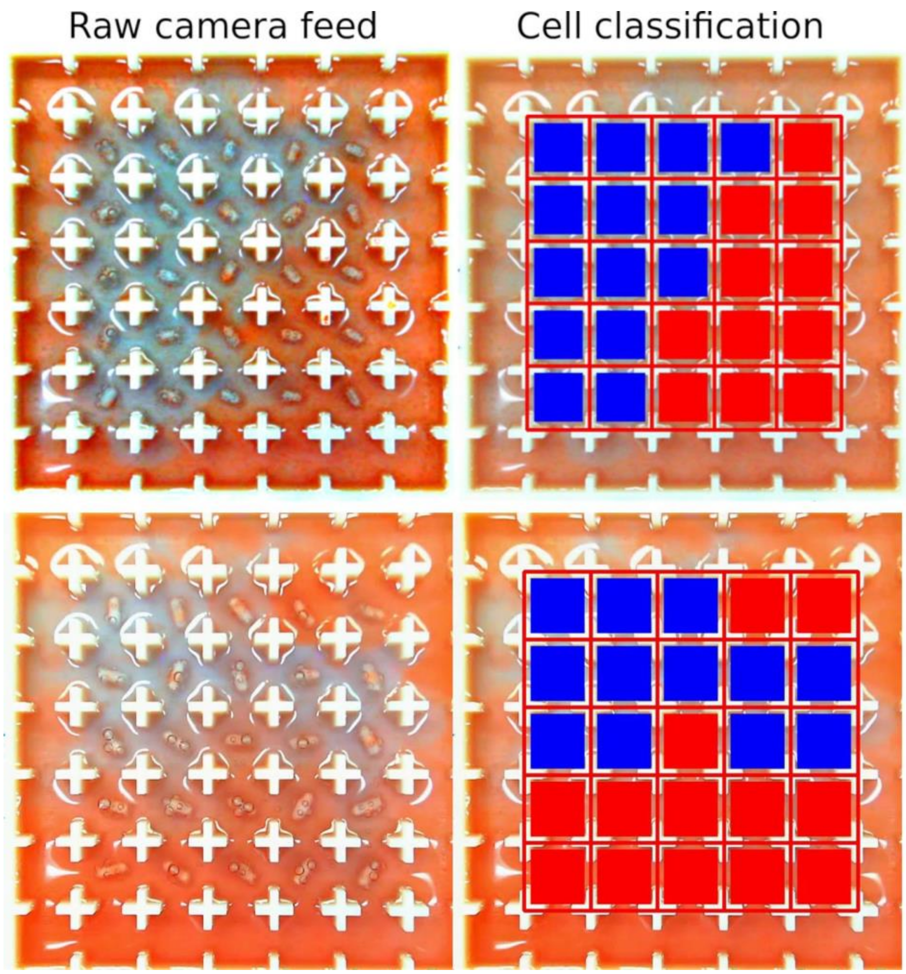

Supplementary Figure 21: Left: Raw camera frame as received by the software. Right: Same frame with the oscillations detected.

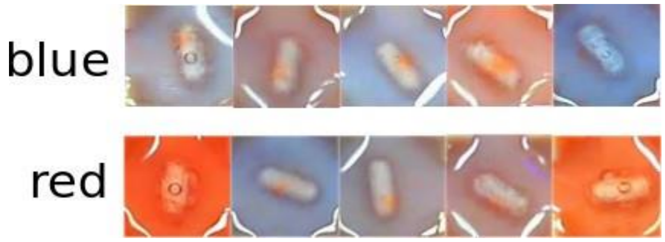

Supplementary Figure 22: Examples of red and blue BZ oscillations as labelled by a human researcher.

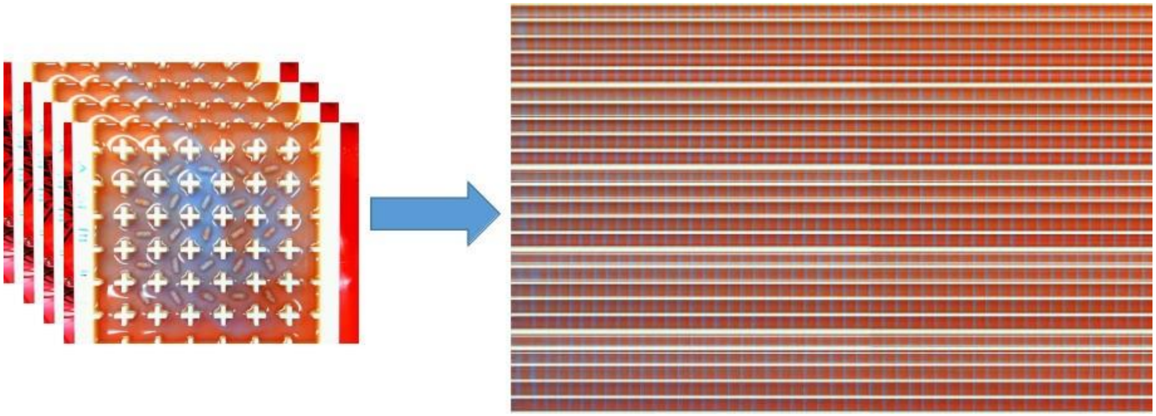

Supplementary Figure 23: Left: A video sequence of BZ reaction. Right: The time map generated, where the blue lines show when the BZ reaction oscillated between states. The ‘y’ axis is the stack of columns from the array lined one above on each other, and the ‘x’ axis is time with each different image showing a different time state for the entire arena.

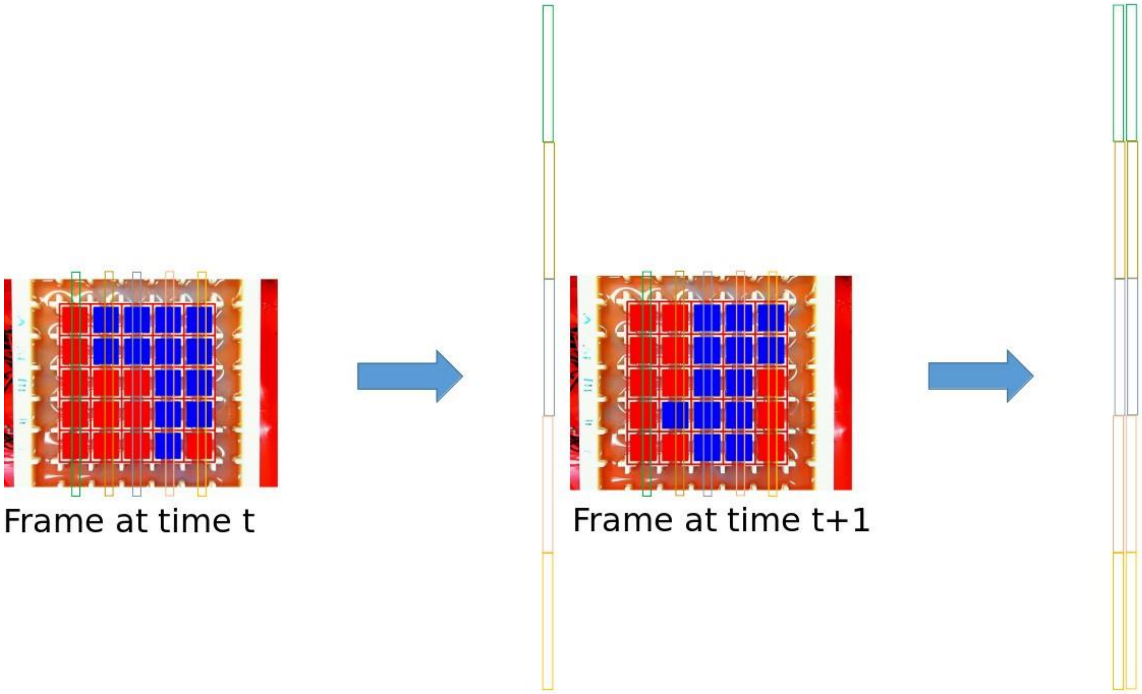

Supplementary Figure 24: Example of how the time-maps were built. The columns used were of 1 pixel width.

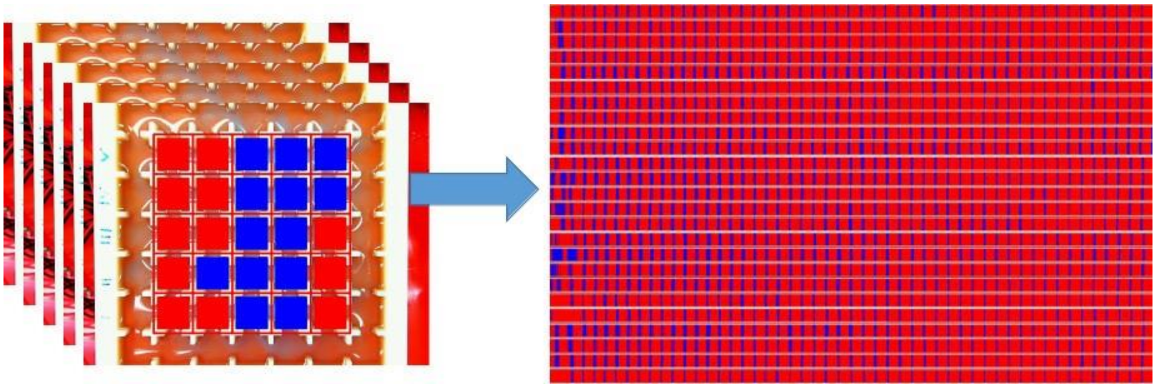

Supplementary Figure 25: Left: A video sequence of BZ reaction. Right: The time map generated, where the blue flashes show when the BZ reaction oscillated. In this case, the video input is a segmented one, and therefore the output also shows a segmented time-map.

3 Chemistry

3.1 Preparation of the reagent solutions for the BZ reaction

The solutions were prepared the following way:

- Ferroin Indicator: A stock solution of 1.0 M was prepared by dissolving 5.406g of 1,10-phenanthroline in 10mL water and adding 2.60g ferrous sulfate hexahydrate while stirring. This stock solution was diluted to 0.1 M. Finally, 15mL of this product were diluted in 95mL of water for a total volume of 110mL.
- Potassium Bromate: 0.5 M solution was prepared by dissolving 8.35g $KBrO_3$ in 100mL of 1 M H_2SO_4.
- Sulfuric Acid: 1 M H_2SO_4 was prepared by taking 5.6mL conc. H_2SO_4 (96%, 18 M) and adding water to reach a total volume of 100mL.
- Malonic Acid: 1 M solution was prepared by dissolving 10.4g malonic acid in 100mL of water.

1 M H_2SO_4 was sourced from Fisher Scientific, >95% analytical reagent grade. Malonic acid was sourced from Sigma Aldrich, Reagent Plus 99%. Ferrous sulfate heptahydrate was sourced from Sigma Aldrich, >=98%. 1,10-phenanthroline was sourced from Sigma Aldrich, >=99%. Potassium bromate was sourced from different sources. The main body of work of this research used the one sourced from Lancaster, 99%. Eventually all of it was used, and we could not source more from the same company. We then sourced it from different suppliers: Scientific Laboratory Supplies, 99% Alfa Aesar, 99.8% Alfa Aesar and Millipore Ensure.

3.2 Experimental protocol

The first step required was to define the working recipe that would be used in the experiments unless stated otherwise. In order to do so, different experiments with different ratios of the components described above

were performed. The objective was to obtain a recipe that would output a constant colour through the time of 1 hour, as well as well-defined oscillations. The results of this search can be seen on Section 4.3. The recipe chosen contained 2.5mL of the ferroin solution, 20mL of water, 12.5mL of the Sulfuric acid solution, 18mL of the malonic acid solution, and finally 19mL of the Potassium bromate solution. Based on this recipe, the experimental protocol is as follows:

1. Prepare the described recipe, in the described order, in a 100mL glass beaker. This glass beaker must contain a stir bar.
2. Place the beaker in a magnetic stirrer. In our case, we used a “IKA big-squid ocean” set to around 33% of speed.
3. Eventually the BZ medium will start to flash or oscillate. Once it flashes clearly twice, transfer it to the arena (motors disabled).
4. Wait for 10 minutes and let the BZ medium rest. The objective is to stop the possible oscillations that were started when the liquid was transferred.
5. Activate the motors in the desired pattern.
6. Record the experiment for 30 minutes (or any other desired length, in our case most of the experiments were 30 minutes).
7. Remove the contents of the arena and clean the arena with water. Wait until all remains of water are gone before starting a new experiment.

The different volumes were transferred using a “SciPette Autoclaveable Variable Pipettor” with a range from 2mL to 10mL, and 0.1mL precision.

4 Experimental data

This section will cover all the experimental data generated using our BZ platform. The main data structure used to show the data will be the time-map plots described on Section 3.3.

4.1 Studying the relation between arena architecture and oscillations

By using 3D printing to construct grids of discrete but fluidically-connected cells, we were able to control the propagation of wave patterns from a cell to a neighbouring cell. The designs were manufactured in a chemically resistant white plastic to allow the colour of each cell to be easily monitored. The basic starting design for the reactionware comprised a monolithic rectangular block into which a square arena was inset. This was then divided into equal sized cells by printing walls at equal spacings, with cut-outs in each wall to allow fluidic connection.

We first performed several pilot experiments to determine a suiSupplementary Table cell design that would produce sustained bulk BZ oscillation waves over the whole cell, while being sufficiently large to be imaged consistently. The best results were obtained using a 7 by 7 grid of 16 by 16 by 5 mm cells where the cut-out accounted for a third of each connecting wall. Of the 7 by 7 grid, only the middle 5 by 5 cells were used for experiments in order to avoid cells on the edges (with fewer neighbours) which might be expected to oscillate differently.

To control the interaction between cells, we first designed a prototype array of BZ cell grid which had a “v” shape opening between cells (Supplementary Figure 26C left) and the BZ reaction volume used was 70 ml, which filled the arena to three quarters of its height, well above the “v” opening. With this design, it was found that oscillations did not propagate to neighbouring cells and the platform acted similarly to a display screen, where only the cells that were enabled flashed in blue, while the other ones remained red (Supplementary Figure 26A-B). This demonstrated that our platform can act as a “display screen”. In order to test it, we used a static activation pattern of cells to flash a “smiley emoji” pattern (Supplementary Figure 26A) and also a sequenced pattern where columns were activated one after the other, left to right (Supplementary Figure 26B).

To facilitate improved interaction between cells, we completely removed the “v” shaped part of the opening, leaving only the corners of each cell to define it (Supplementary Figure 26C right) as fluid dynamics simulations showed that the removal of the barriers allowed the fluid from a stirred cell propagate to neighbouring cells upon stirring. This way, as can be seen in the 4-way neighbourhood test, the fluid from an activated cell would propagate to its neighbours when stirred, and we could, for example, activate a cell that was disabled by stirring (and therefore activating) its neighbours.

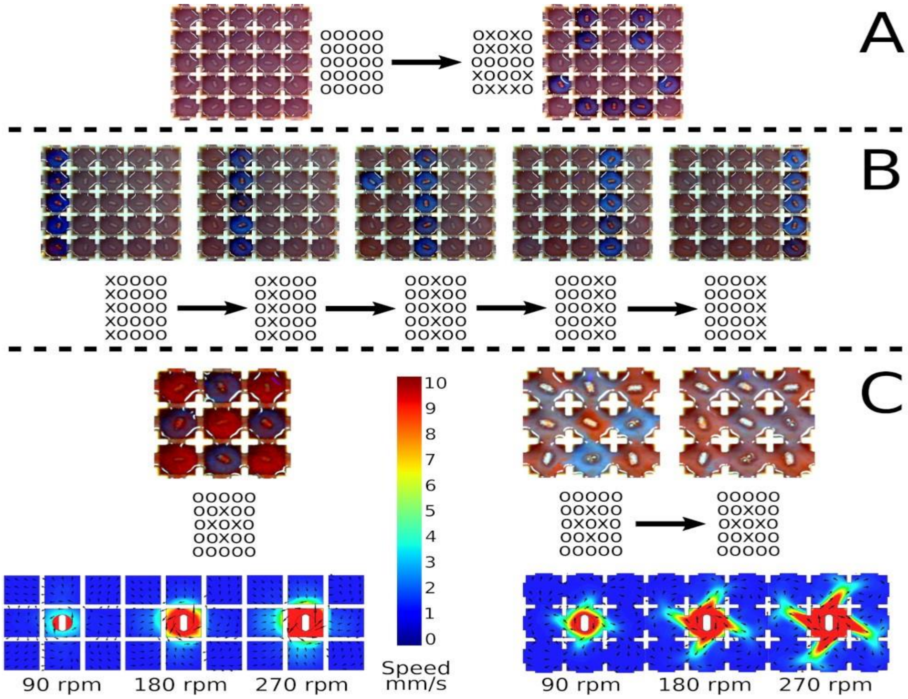

Supplementary Figure 26: Addressing the oscillating cells. In this Supplementary Figure, every video frame is accompanied by a matrix of “x” and “o”. This matrix indicates if in that given frame a motor was enabled (marked with a “x”) or disabled (marked with a “o”). In the first experiments, a grid design with 16mm by 16mm cells with 5 mm of depth, in a grid with 7 by 7 cells, and containing 70 ml of BZ reaction was used. Out of those 49 cells, only the 5 by 5 in the centre contained a stir bar and were placed above a motor, thus the outer 24 ones were always disabled. With a v-shape opening between cells, we could flash images like in a display screen. In (a) for example, it was flashed a smiley emoji, while in (b) it was flashed a sequence of patterns were initially only the left most column was enabled, followed by the adjacent columns in successive steps, until arriving to the right most column. The main drawback of this v-shape opening between cells is that very little fluid was transferred between them, as can be seen in C-left. Thus, we decided to completely open the gap, see C-right, and this allowed for bigger quantities of fluid to move between cells, and in this way we achieved the objective of enabling a cell which was disabled just by enabling its surrounding cells.

4.2 Input patterns used for pattern recognition

In all the following Supplementary Figures white square means disabled, while red square means enabled. In some of the experiments, “disabled” meant that the motor was completely off, and “enabled” meant that the motor was on at double speed of the default speed explained during the Firmware section (Section 1.3). This was for example the case of the AND/OR experiments explained later. In some other cases, “disabled” meant that the motor was actuated at the default speed, while “enabled” meant that the motor was actuated at five times the default speed. This was the case of the XOR experiment explained later in this section. All the pattern recognition experiments used this protocol. The reason behind this was about generating global BZ waves, while in the case of “disabled” meaning completely off, the waves were not necessarily global.

Finally, when here we mean “twice the speed” or “five times the speed”, we mean in terms of the PWM signal generated, not in terms of the actual speed of the motors in terms of revolutions per minute.

Supplementary Figure 27 shows the input patterns used for 0 to 9. Number “1” used two different shapes, one was a straight line, while the other had more shape. Supplementary Figure 28 shows the input patterns used for the letters A, B, C, E, G, L, M, N and R. Supplementary Figure 29 shows three input random patterns that were used with five, seven and nine enabled motors.

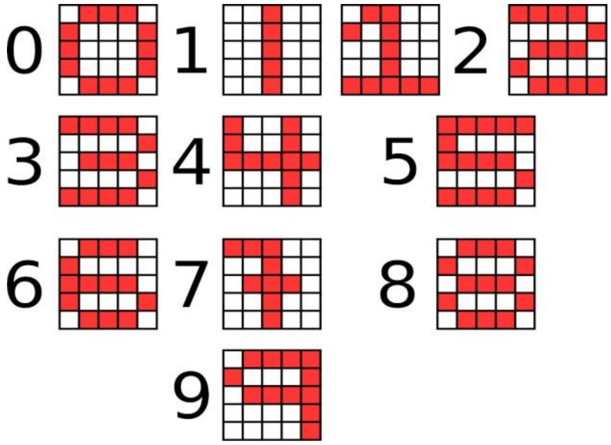

Supplementary Figure S 27: Input patterns used for pattern recognition in the BZ platform. White means disabled, red means enabled. These patters represent the numbers from 0 to 9.

4.3 Studying the colour changes based on the input recipe

The BZ system used during this research, as explained in Section 2, oscillates between two states, which for simplicity we named “red” and “blue”. The BZ medium changes colour as the BZ reaction goes on. Initially it has an almost purple colour, with the oscillations being blue, while after one hour it has a dark red colour, with the oscillations being dark blue. Eventually, the medium is dark red, and no oscillations can be seen. In order to help detecting the oscillations as explained in the image processing section (Section 3), we tried to utilize a BZ recipe in which the colour changed the least in a 1 hour long experimental window. This section will show the different recipes used and the colour variation they produced. The one chosen was: 2.5 mL Ferroin, 20 mL water, 12.5 mL sulfuric acid, 18 mL malonic acid and 19 mL potassium bromate. The only reason this recipe was chosen is because it produced the most constant colour scheme. It is also worth noting that different recipes will produce different regimes of oscillations, and when choosing between different recipes that looked potentially good (from a colour scheme perspective) we settled for the one with the highest frequency of oscillations.

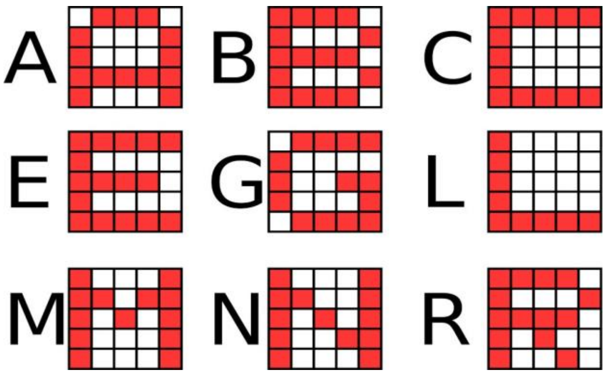

Supplementary Figure S 28: Input patterns used for pattern recognition in the BZ platform. White means disabled, red means enabled. These patterns represent the letters A, B, C, E, G, L, M, N and R.

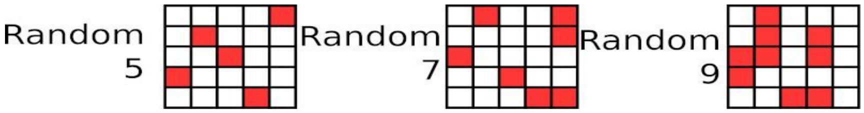

Supplementary Figure 29: Input patterns used for pattern recognition in the BZ platform. White means disabled, red means enabled. These patterns represent three different patterns that were randomly chosen with 5, 7 and 9 enabled motors.

In all the following Supplementary Figures, the BZ medium was prepared and placed using the protocol described in Section 2, and then all the motors were switched on at the default speed for 1 hour. Supplementary Figures 30 to 40 show the results.

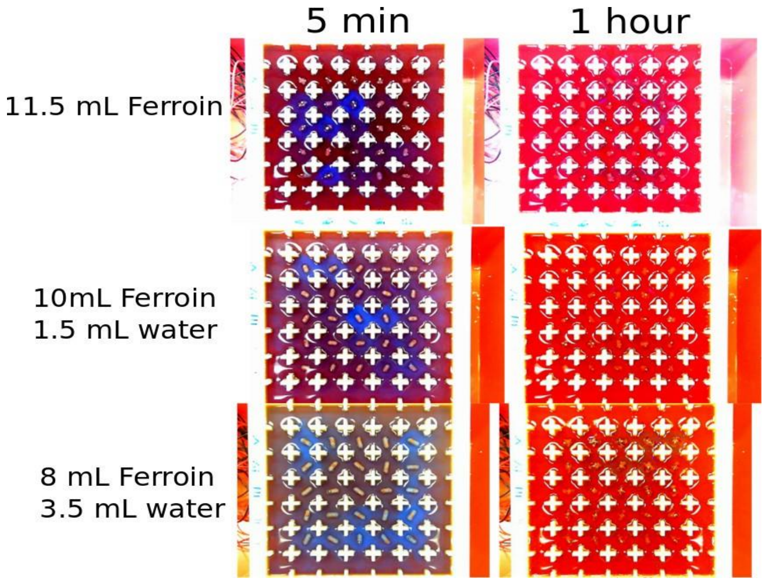

Supplementary Figure 30: Testing for the BZ recipe with the best colour scheme. In this case, none of these recipes produced flashes after 1 hour. The recipe here used contains 15 mL sulfuric acid, 21.5 mL malonic acid and 22.5 mL potassium bromate. First row of the image then contains 11.5 mL of Ferroin, the second one 10 mL of Ferroin and 1.5 mL of water, and the last row 8 mL of Ferroin and 3.5 mL of water.

The objective was to obtain the least possible change in colour. Good candidates were identified, and for each of them a time-map (as explained in Section 3.3) was generated. As an example of a experimental recipe with a big change of colour, Supplementary Figure 41 is shown. Supplementary Figures 41 to 46 show the time-map of the main candidates. It can be seen that some of them were not able of producing oscillations through the whole experimentation time. The last one in particular: 2.5mL ferroin,12.5mL sulfuric acid, 18mL malonic acid, 19mL potassium bromate and 20mL of water; was the main recipe used during this research.

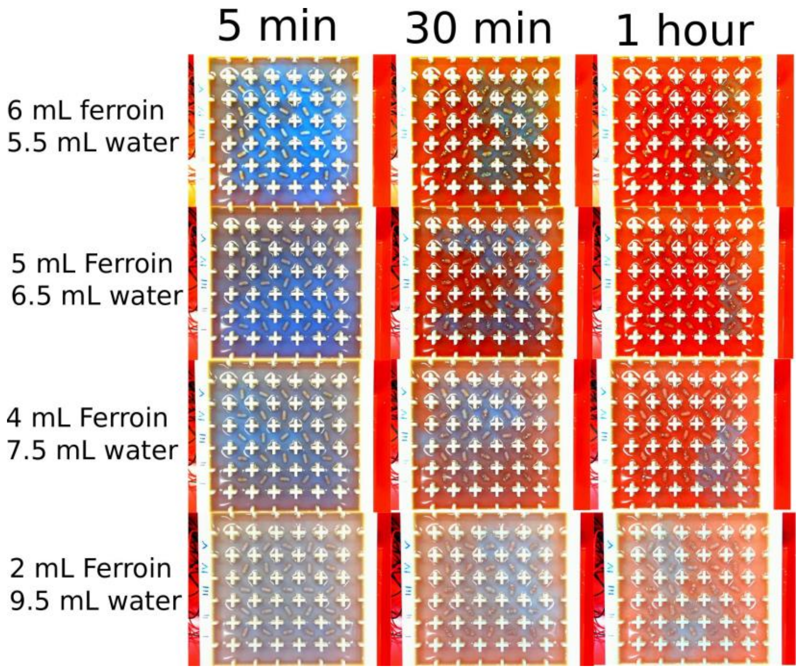

Supplementary Figure 31: Testing for the BZ recipe with the best colour scheme. The recipe here used contains 15 mL sulfuric acid, 21.5 mL malonic acid and 22.5 mL potassium bromate. First row of the image then contains 6 mL of Ferroin and 5.5 mL of water, the second 5 mL of Ferroin and 6.5 mL of water, the third one 4 mL Ferroin and 7.5 mL water, and the last row 2 mL of Ferroin and 9.5 mL of water.

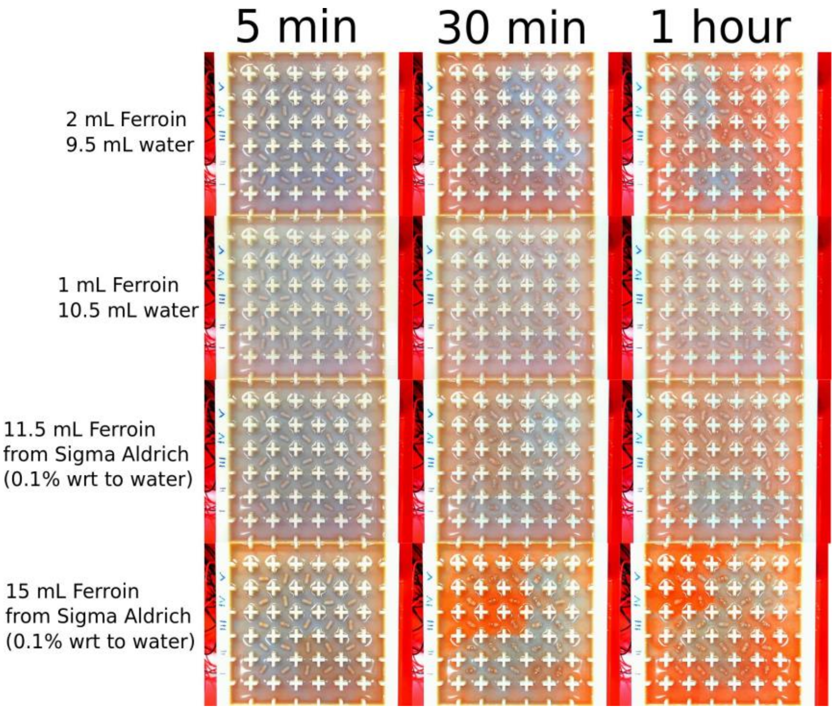

Supplementary Figure 32: Testing for the BZ recipe with the best colour scheme. The recipe here used contains 15 mL sulfuric acid, 21.5 mL malonic acid and 22.5 mL potassium bromate. First row of the image then contains 2 mL of Ferroin and 9.5 mL of water, the second 1 mL of Ferroin and 10.5 mL of water, the third one 11.5 mL Ferroin sourced from Sigma (0.1% wrt to Water), and the last row 15 mL Ferroin sourced from Sigma (0.1% wrt to Water).

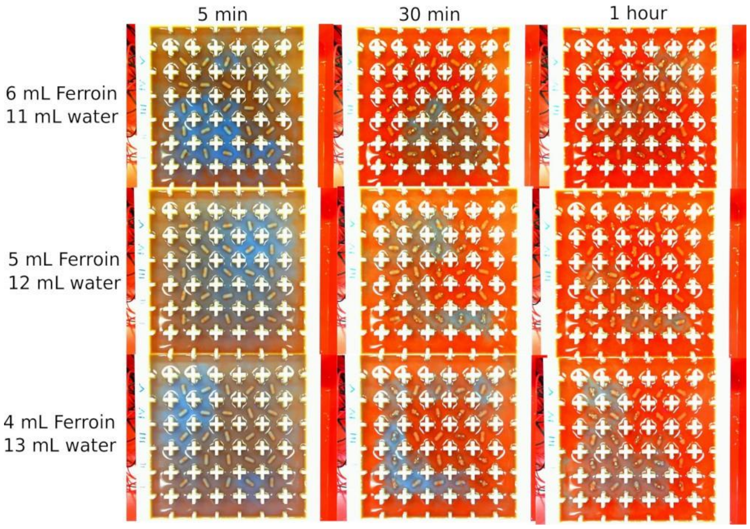

Supplementary Figure 33: Testing for the BZ recipe with the best colour scheme. The recipe here used contains 13.5 mL sulfuric acid, 19.5 mL malonic acid and 20.5 mL potassium bromate. First row of the image then contains 6 mL of Ferroin and 11 mL of water, the second 5 mL of Ferroin and 12 mL of water, and the last row 4 mL of Ferroin and 13 mL of water.

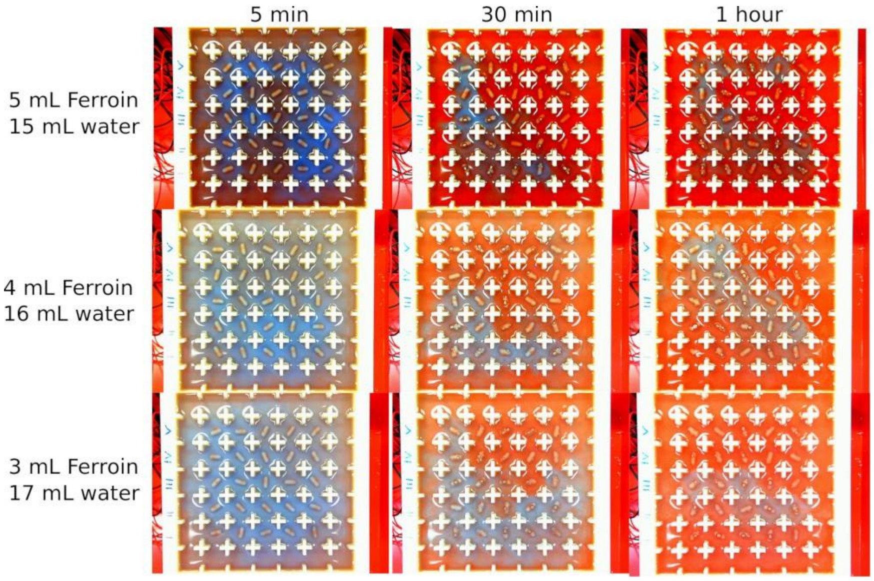

Supplementary Figure 34: Testing for the BZ recipe with the best colour scheme. The recipe here used contains 12.5 mL sulfuric acid, 18.5 mL malonic acid and 19.5 mL potassium bromate. First row of the image then contains 5 mL of Ferroin and 15 mL of water, the second 4 mL of Ferroin and 16 mL of water, and the last row 3 mL of Ferroin and 17 mL of water.

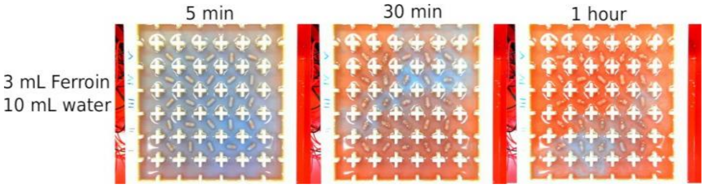

Supplementary Figure 35: Testing for the BZ recipe with the best colour scheme. The recipe here used contains 15.5 mL sulfuric acid, 21.5 mL malonic acid and 23.5 mL potassium bromate. The only row contains 3 mL Ferroin and 10 mL of water.

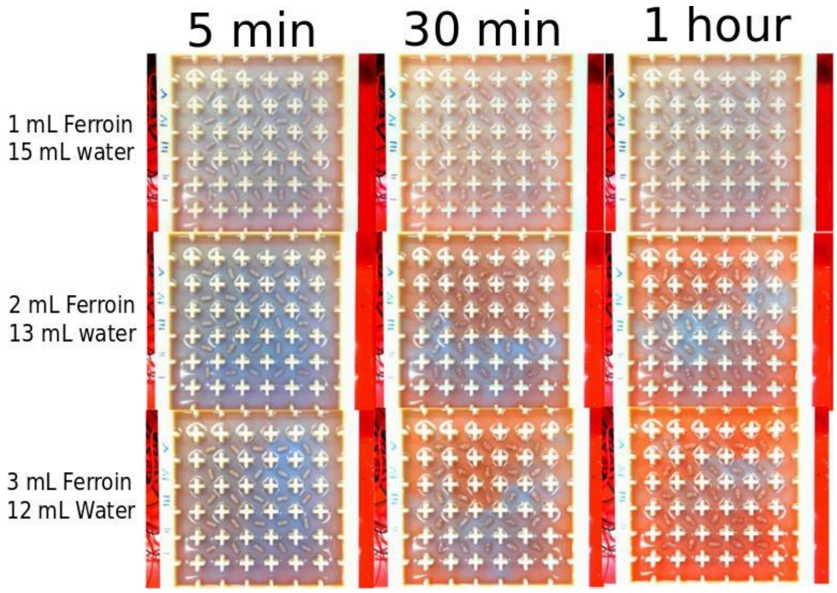

Supplementary Figure 36: Testing for the BZ recipe with the best colour scheme. The recipe here used contains 15 mL sulfuric acid, 21.5 mL malonic acid and 22.5 mL potassium bromate. The first row contains 1 mL Ferroin and 14 mL of water, the second row contains 2 mL Ferroin and 13 mL water, the third row contains 3 mL Ferroin, 12 mL water.

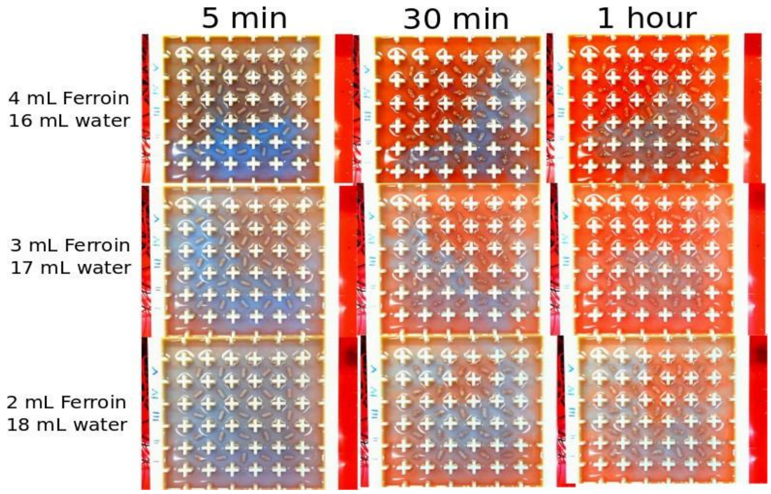

Supplementary Figure 37: Testing for the BZ recipe with the best colour scheme. The recipe here used contains 15 mL sulfuric acid, 21.5 mL malonic acid and 22.5 mL potassium bromate. The first row contains 4 mL Ferroin and 16 mL of water, the second row contains 3 mL Ferroin and 17 mL water, the third row contains 2 mL Ferroin, 18 mL water.

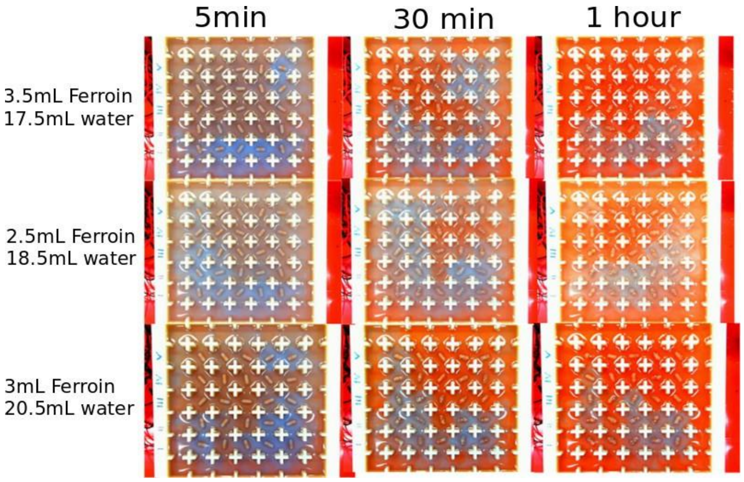

Supplementary Figure 38: Testing for the BZ recipe with the best colour scheme. The recipe here used contains 12.5 mL sulfuric acid, 18 mL malonic acid and 19 mL potassium bromate. The first row contains 3.5 mL Ferroin and 17.5 mL of water, the second row contains 2.5 mL Ferroin and 18.5 mL water, the third row contains 3 mL Ferroin, 20.5 mL water.

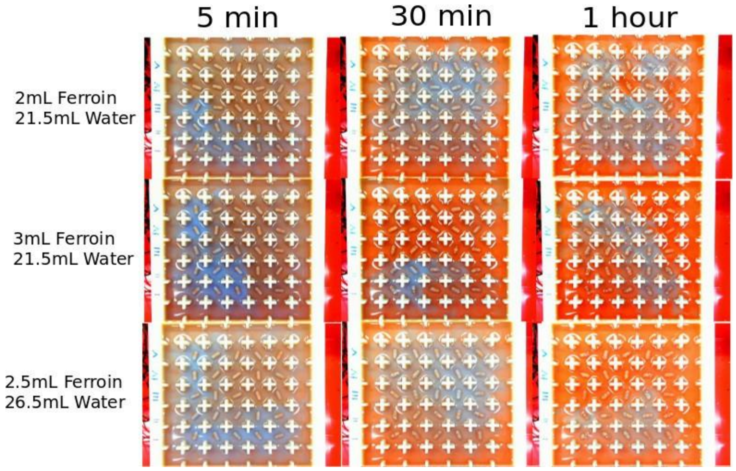

Supplementary Figure 39: Testing for the BZ recipe with the best colour scheme. The recipe here used contains 12.5 mL sulfuric acid, 18 mL malonic acid and 19 mL potassium bromate. The first row contains 2 mL Ferroin and 21.5 mL of water, the second row contains 3 mL Ferroin and 21.5 mL water, the third row contains 2.5 mL Ferroin, 26.5 mL water.

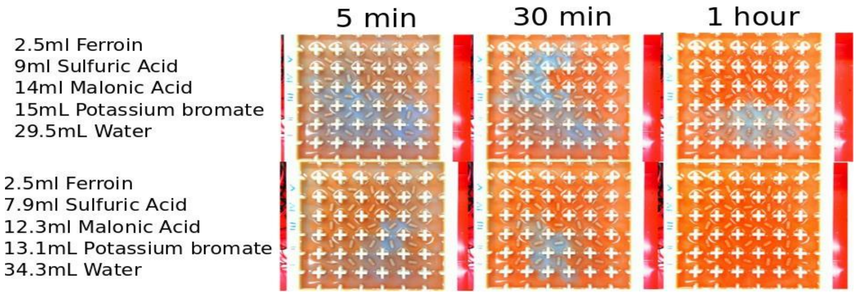

Supplementary Figure 40: Testing for the BZ recipe with the best colour scheme. The two rows have different recipes, as shown by the legend.

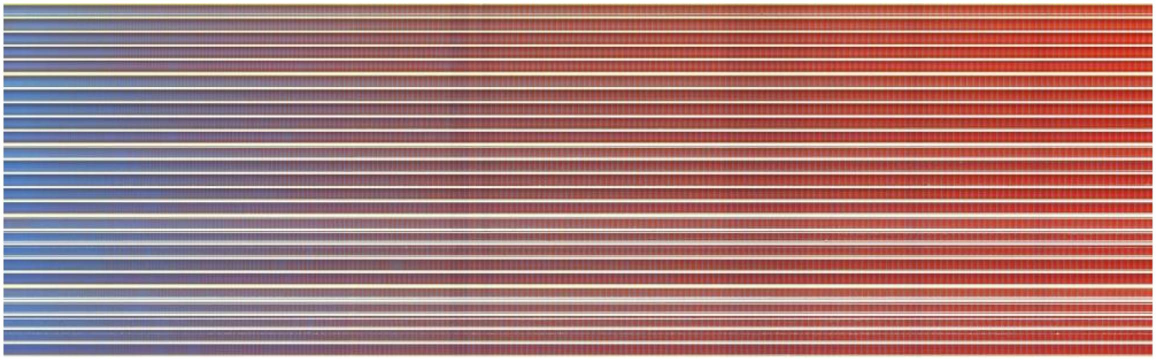

Supplementary Figure 41: One hour time-map for the recipe containing 4mL of Ferroin, 10mL of sulfuric acid, 15mL of malonic acid, 21.5mL of potassium bromate and 22.5mL of water.

4.4 Reaction-diffusion experiments in a device without walls

An experimental arena like the one shown on Supplementary Figure 5 was tested. This arena did not contain walls or corners. The results can be seen on Supplementary Figure 47. In this experiment, all the stirrers were "on" at the default speed (90 RPM). The experiment lasted 2 minutes and 36 seconds.

4.5 Testing for how long the BZ system can oscillate

Three-hour long experiments were performed to test for how long the BZ reaction would oscillate. All the motors were set to "on" to the default speed, and the BZ medium continued to oscillate until the three-hour mark, see Supplementary Figure 48.

4.6 Studying the memory effect of the BZ medium

A very interesting phenomena of the BZ medium is that it has memory. That is, if a pattern of motors is enabled and therefore their stir bars rotate, a global wave will emerge in the system. If these motors then are disabled, this global wave will continue in the system for a series of oscillations, until it finally disappears.
This was tested in a series of experiments, where all the motors were first enabled, and then disabled, in continuous cycles. The total length of these experiments was 1 hour. First, the motors were enabled and disabled in cycles of 5 minutes (5 minutes on, followed by 5 minutes off), see Supplementary Figure 49. Therefore, in 1 hour there were 6 of these on/off cycles. The oscillations seemed to perfectly persist after 5 minutes of all the motors being disabled. Secondly, a similar experiment was done, but in this case the motors were on for 5 minutes, followed by 10 minutes of them being disabled, see Supplementary Figure 50.

Therefore, in one hour there were four of these on/off cycles. In this experiment it can be seen that after 5 minutes of all the motors being off, the signal starts to weaken, and around the 10 minutes mark, when the motors are re-started, the signal was almost completely gone. This indicates that the system can keep a memory for around 5 minutes, although more experiments would be needed in order to certify the exact time

the memory can keep a signal. One very interesting thing about this phenomena, is that each BZ cell can be considered like a cell in a recurrent neural network (RNN). Following the RNN model, we outlined the BZ process on Supplementary Figure 51, where it can be seen how the state depends on the inputs, the state of the neighboring cells, and the previous global state of the BZ medium.

In order to quantify the relation between the BZ chemical recipe and the “memory” of the oscillations, it was decided to perform a series of experiment using the default BZ recipe but changing the quantity of $KBrO_3$ from 12 to 22 ml in steps of 1ml. In this particular experiment, as soon as it starts all the stirrers are enabled for 7.5 minutes. Then all of them are disabled for 7.5 minutes. Then all of them are enabled for 7.5 minutes. Finally, all of them are disabled for 7.5 minutes. The results can be seen on Supplementary Figure 52. These results seem to indicate that the number of oscillations once the stirrers are stopped (the “memory”) increase with the quantity of $KBrO_3$. This might be perhaps related with the fact that a higher concentration of $KBrO_3$ generates oscillations with a higher frequency.

4mL ferroin, 13.5mL H2SO4, 19.5mL malonic acid, 20.5mL KBrO3, 13mL water

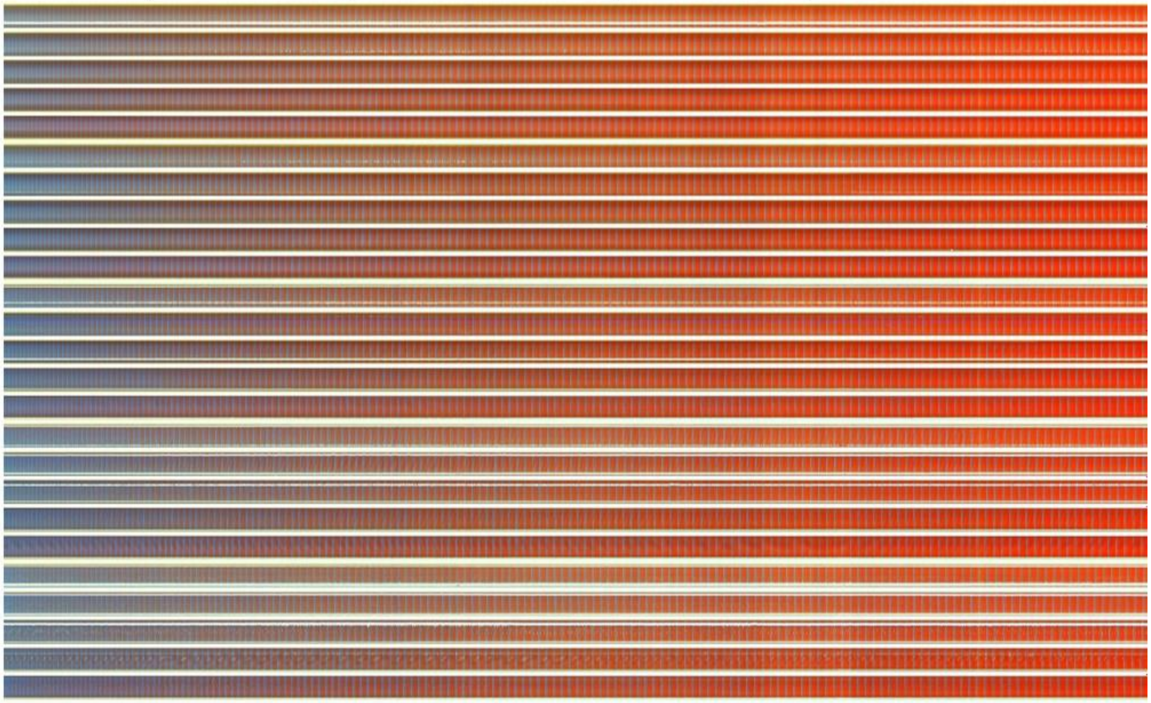

3.5mL ferroin, 12.5mL H2SO4, 18mL malonic acid, 19mL KBrO3, 17.5mL water

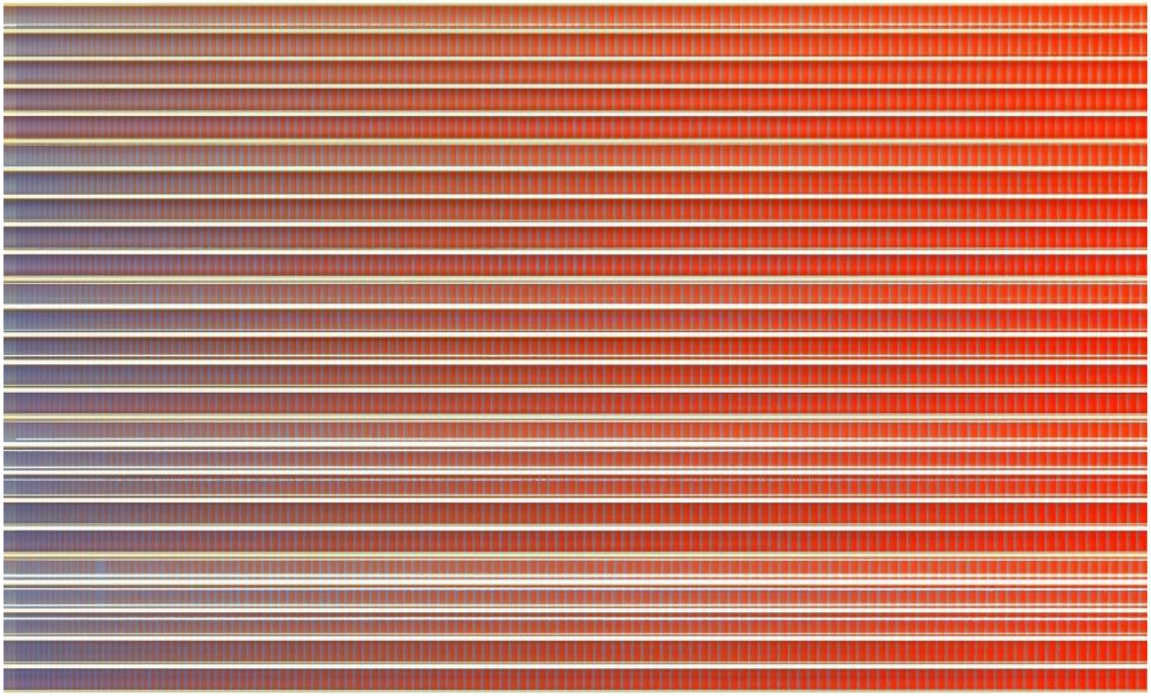

Supplementary Figure 42: One hour time-map for two of the best colour-scheme candidates. In this case the focus was in the oscillations.

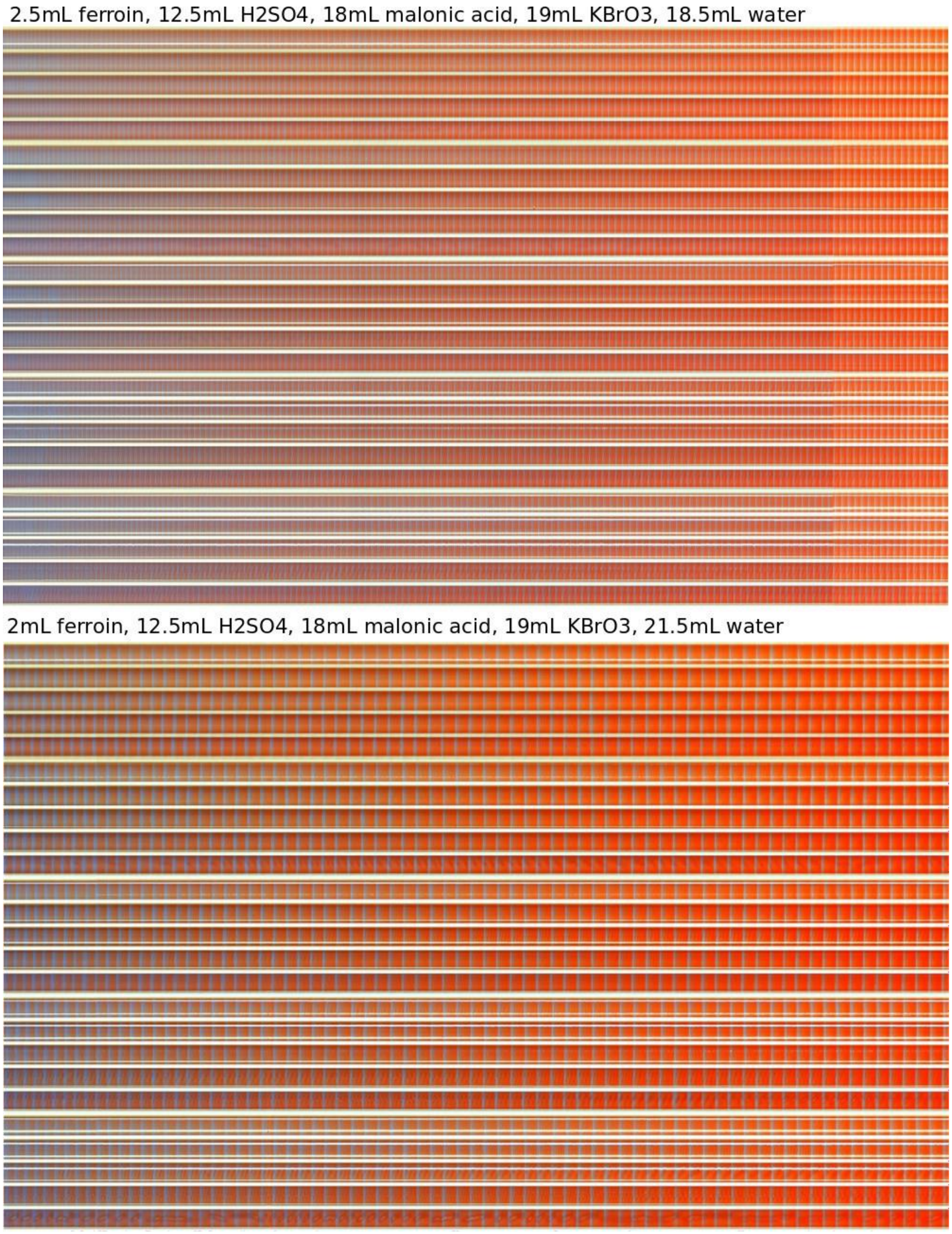

Supplementary Figure 43: One hour time-map for two of the best colour-scheme candidates. In this case the focus was in the oscillations.

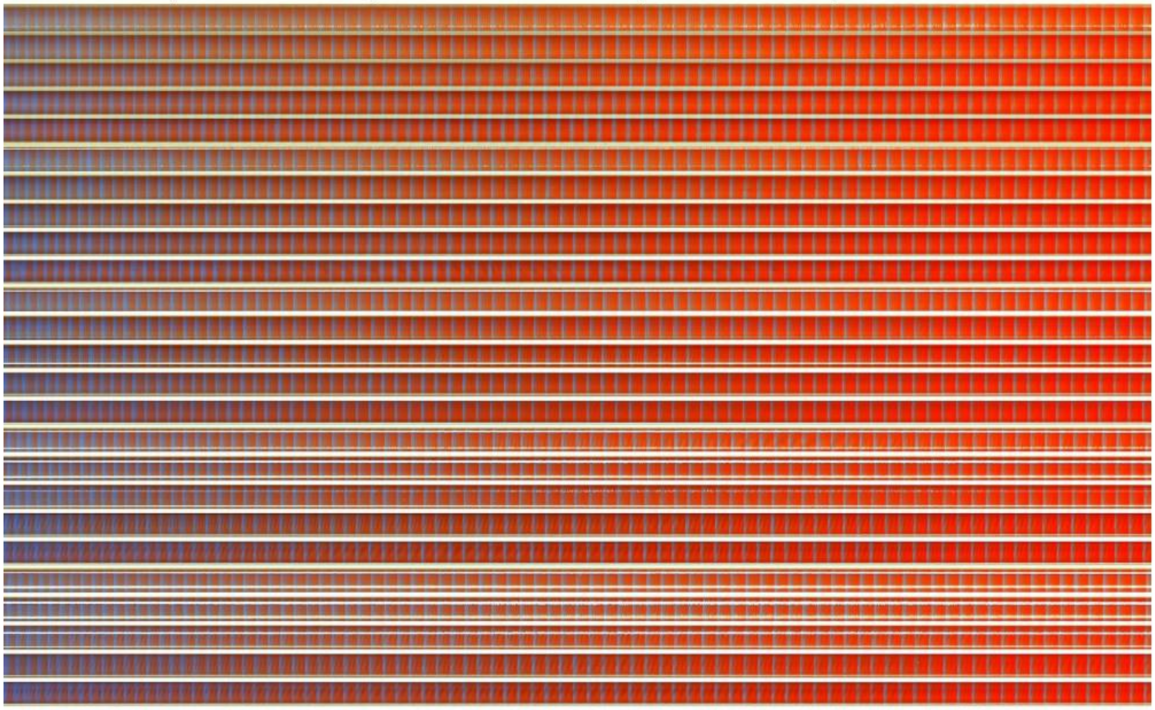

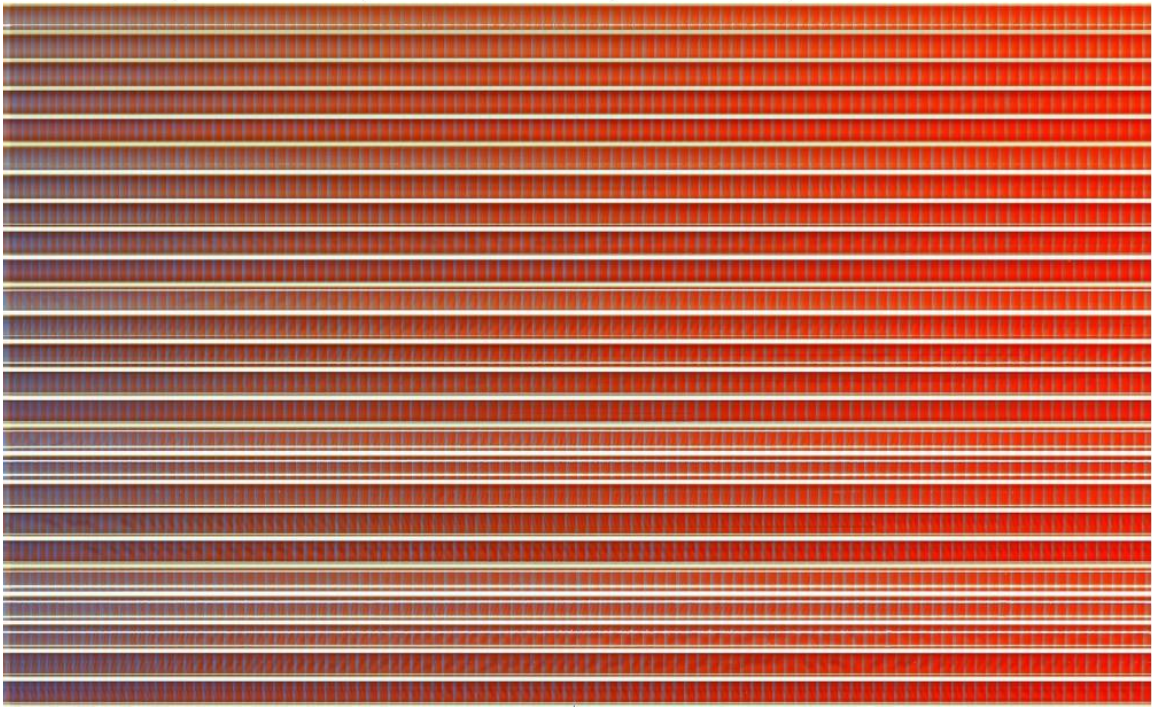

Supplementary Figure 44: One hour time-map for two of the best colour-scheme candidates. In this case the focus was in the oscillations.

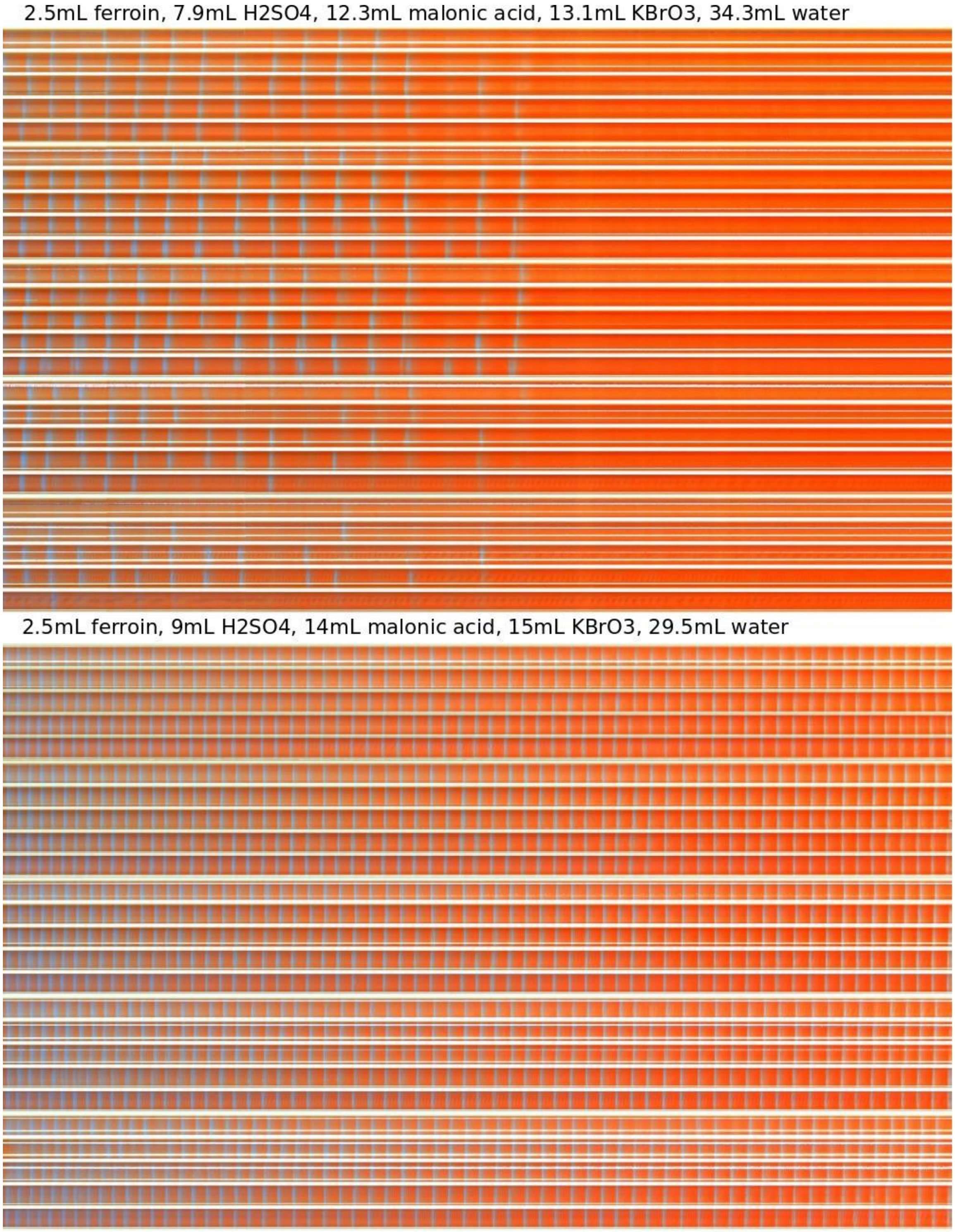

Supplementary Figure 45: One hour time-map for two of the best colour-scheme candidates. In this case the focus was in the oscillations.

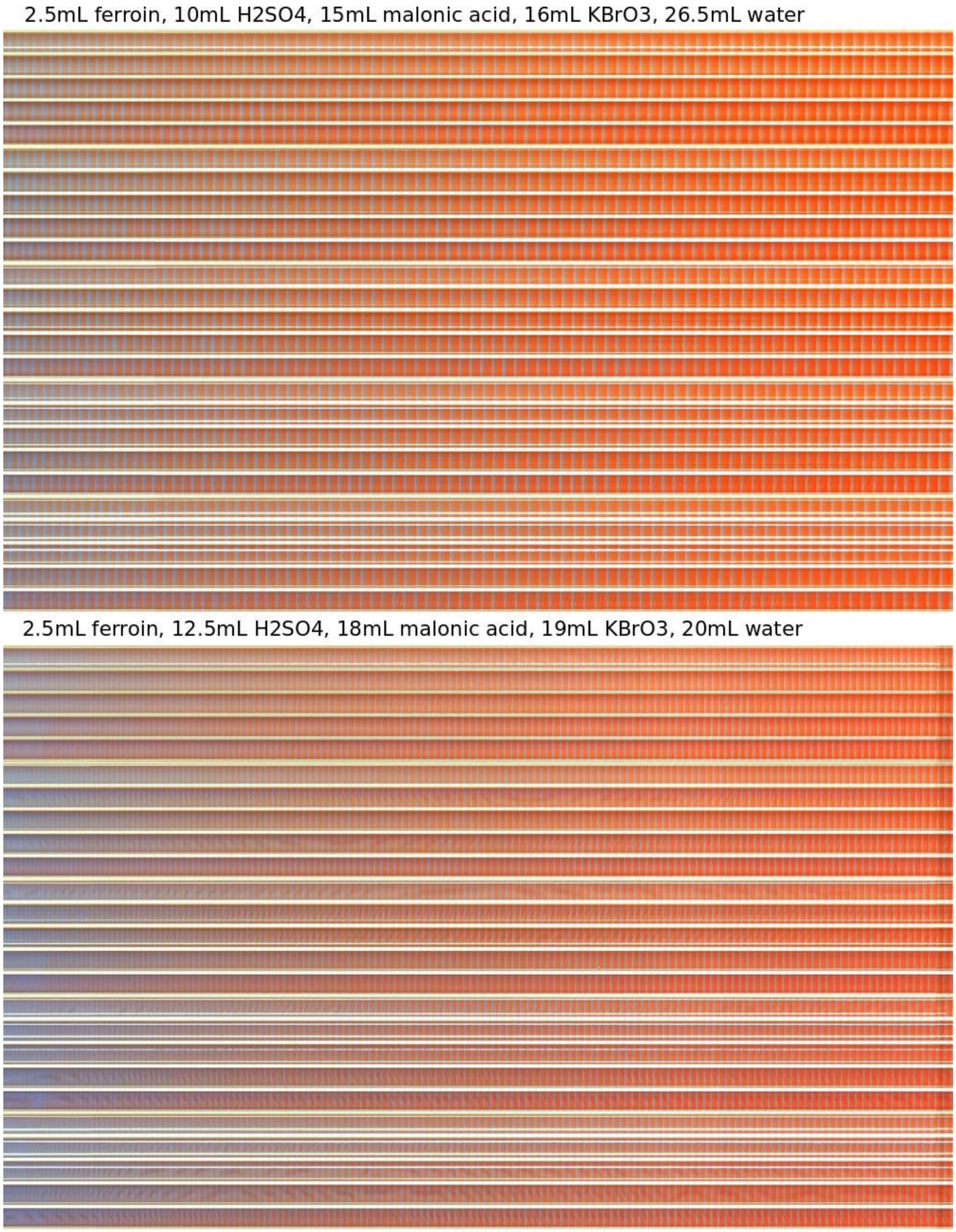

Supplementary Figure 46: One hour time-map for two of the best colour-scheme candidates. In this case the focus was in the oscillations. The second recipe here is the main recipe used during this research: 2.5mL ferroin, 14mL of sulfuric acid, 19mL of potassium bromate, 18mL of malonic acid and 20mL of water.

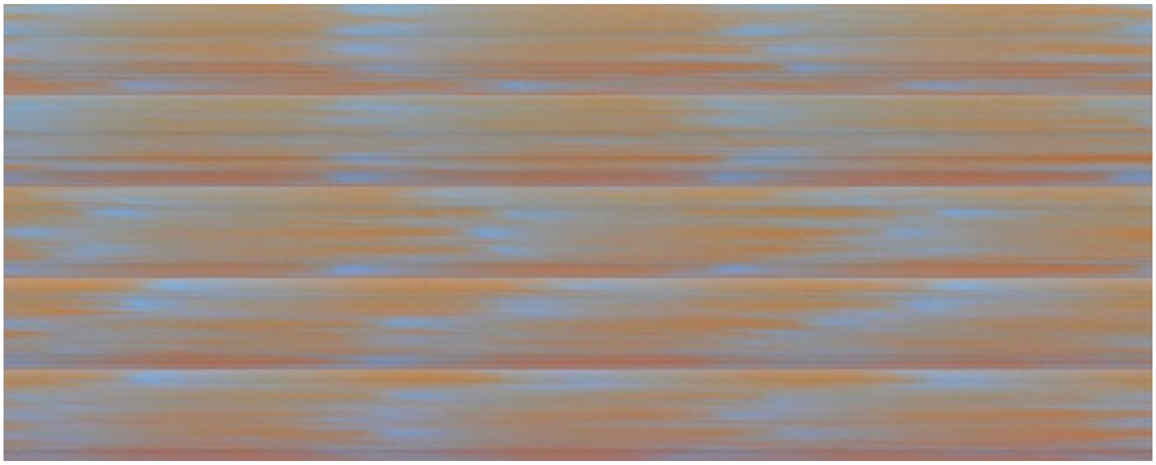

Supplementary Figure S 47: Experiment using a experimental arena without walls (see Supplementary Figure 5). Here the timemap is shown. In total this experiment lasted 2 minutes and 36 seconds. At 30 FPS every row of pixels in this image represents a frame.

4.7 Studying the speed of stir rotation and its relation to BZ oscillations

It was also tested how different stirrer speeds impact the frequency of oscillations. In order to do so, the pattern flashed was “10001” meaning the first column of stirrers were enabled, the following three were completely off (not moving) and the last column was also enabled. The speeds tested were: default speed, two times the default speed, and five times the default speed. Supplementary Figure 53 shows the time-map for the three speeds. In this case we took the first five rows of the time-maps instead of the 25 as in the previous ones. We also only focused on the last 15 minutes of the experiment. We did it this way because it was considered that they would be easier to compare. It can be seen in the results how higher stirring speeds actually decrease the frequency of the oscillations. In these results, the default speed generated 26 oscillations in 15 minutes, double the speed generated 24 oscillations, and five times the speed generated 23 oscillations. These numbers seem very similar, but in the Supplementary Figure described it can better be seen. Another side-effect of high speeds is that they sometimes failed to generate oscillations. This can be seen in the Supplementary Figure described, in the last two rows within the last row: near the end, it fails to oscillate.

Setting each stirrer at a random speed

A series of experiments were performed were the speed of each motor was set randomly. We used Python Random library to choose a number between 0 and 4000 for each motor. As described before, PWM signals under 500 did not produce motor rotations, therefore, some of the motors were disabled. Our expectation was that the system would struggle to arrive to a coherent state pattern, and it did take more than that usual, but it did manage to arrive to a coherent pattern. See Supplementary Figure 54. There it can be seen, for example, that the 21st row (fifth from the bottom) took a third of time to oscillate, but eventually it did oscillate. The total time of this experiment was 30 minutes.

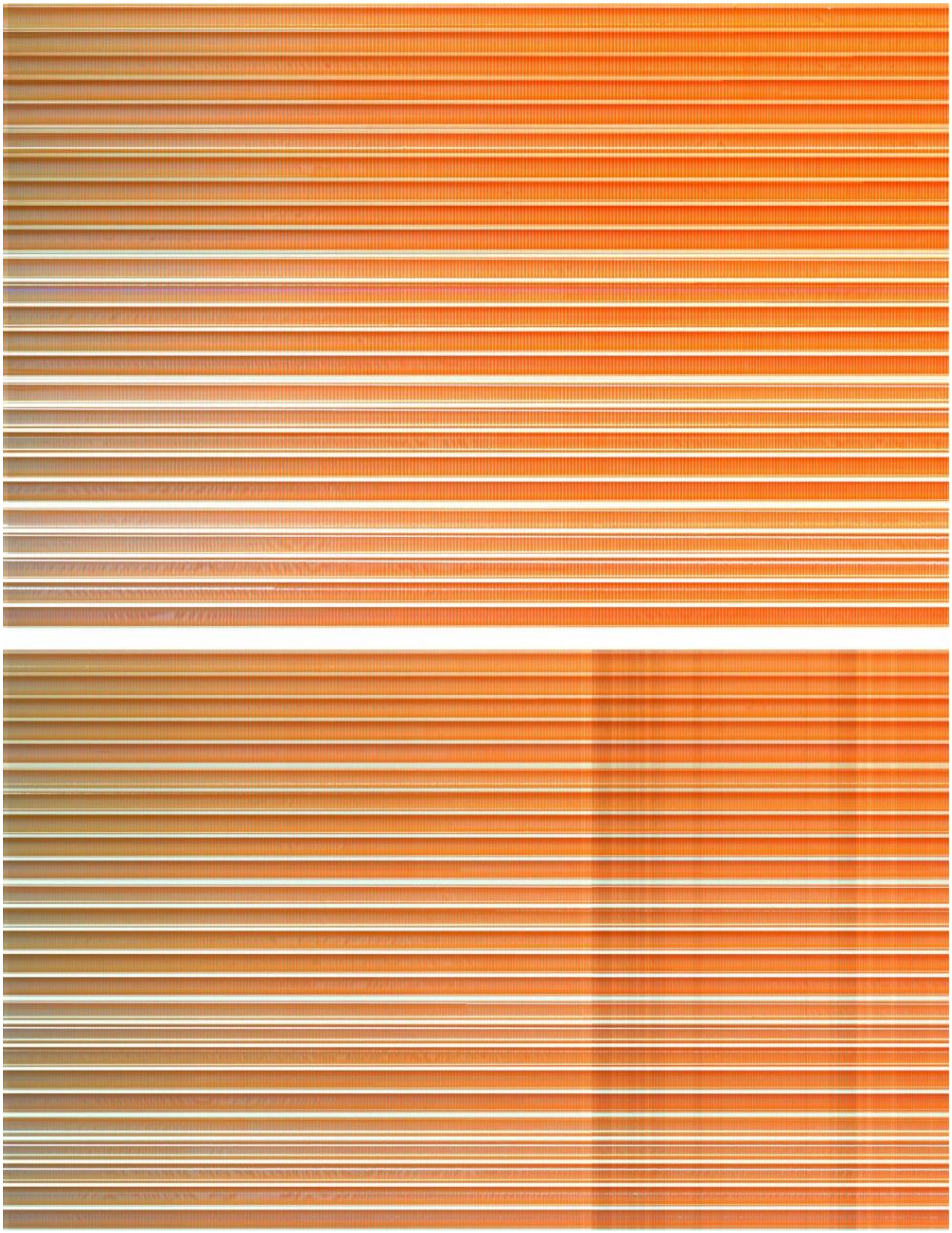

Supplementary Figure 48: Two executions of 3 hour long experiments. All the motors were set to the default speed.

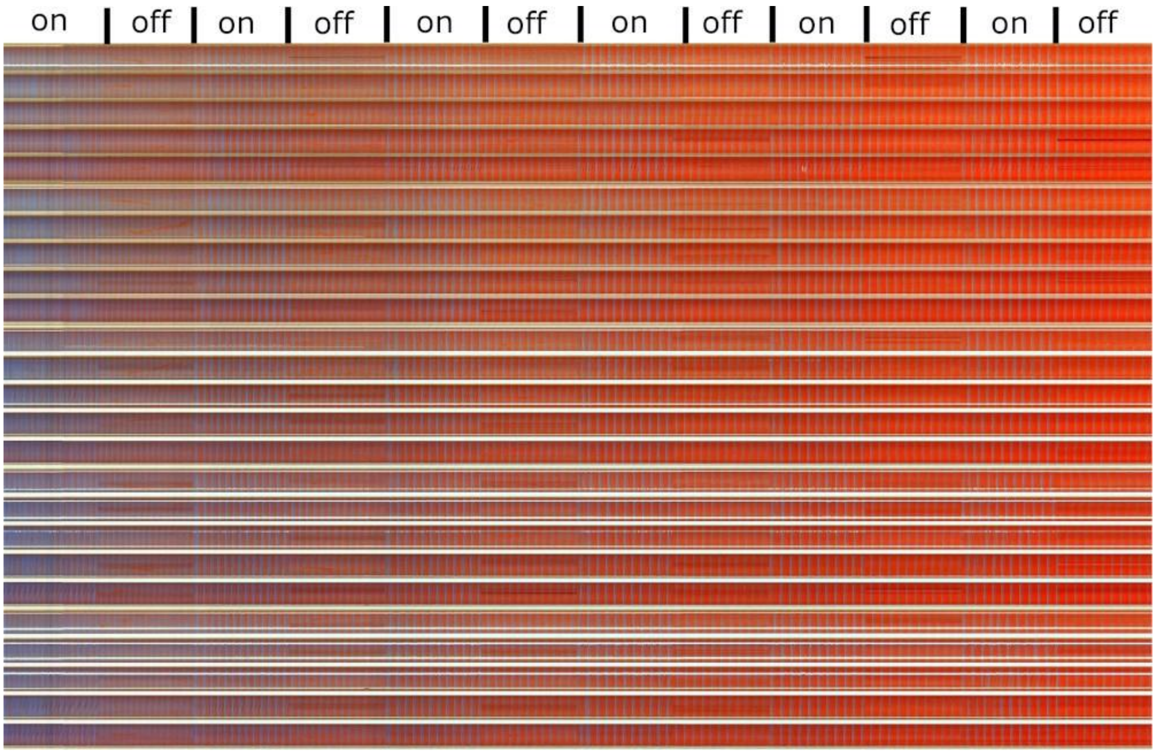

Supplementary Figure 49: Enabling and disabling all the motors in 5 minute cycles. Total experiment was 1 hour.

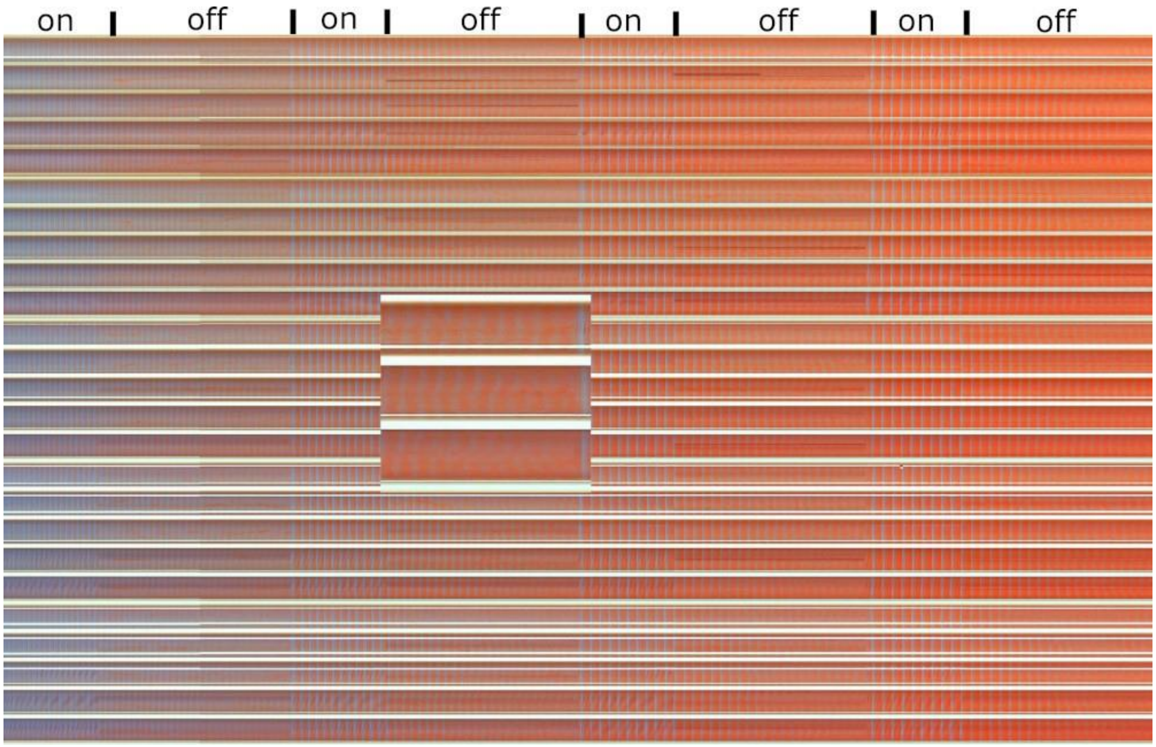

Supplementary Figure 50: Enabling all the motors for 5 minutes, and then disabling them for 10. Total experiment was 1 hour. Centre image zooms into the end of a 10 minutes off period.

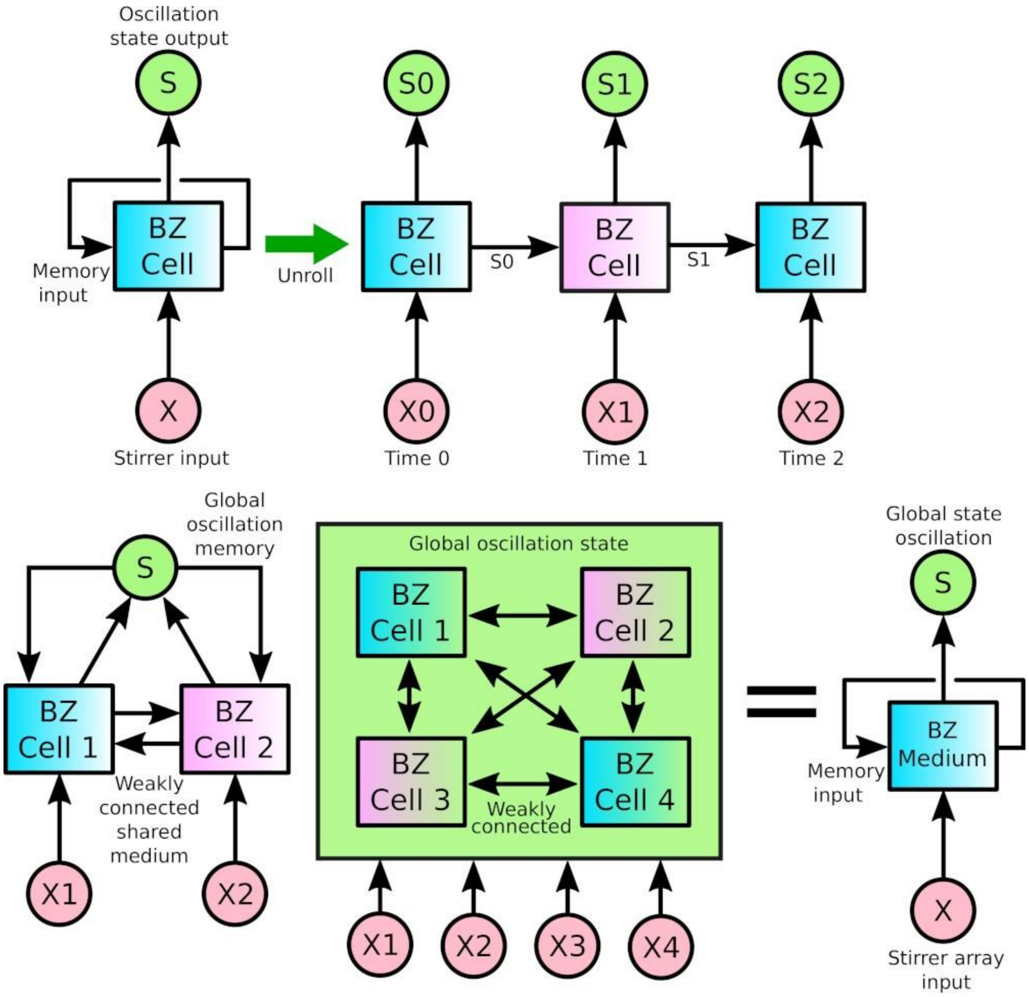

Supplementary Figure 51: In systems with multiple cells weakly connected, not only the state depends on the inputs plus the memory of the system, but also depends on the weakly connections between cells. The top row in this Supplementary Figure represents a system with only one cell. Bottom-left image represents a system with two cells, and bottom-right represent a system with multiple cells. This multiplecell system can be "encapsulated" and seen like the system with only one cell in terms of inputs, memory, and output.

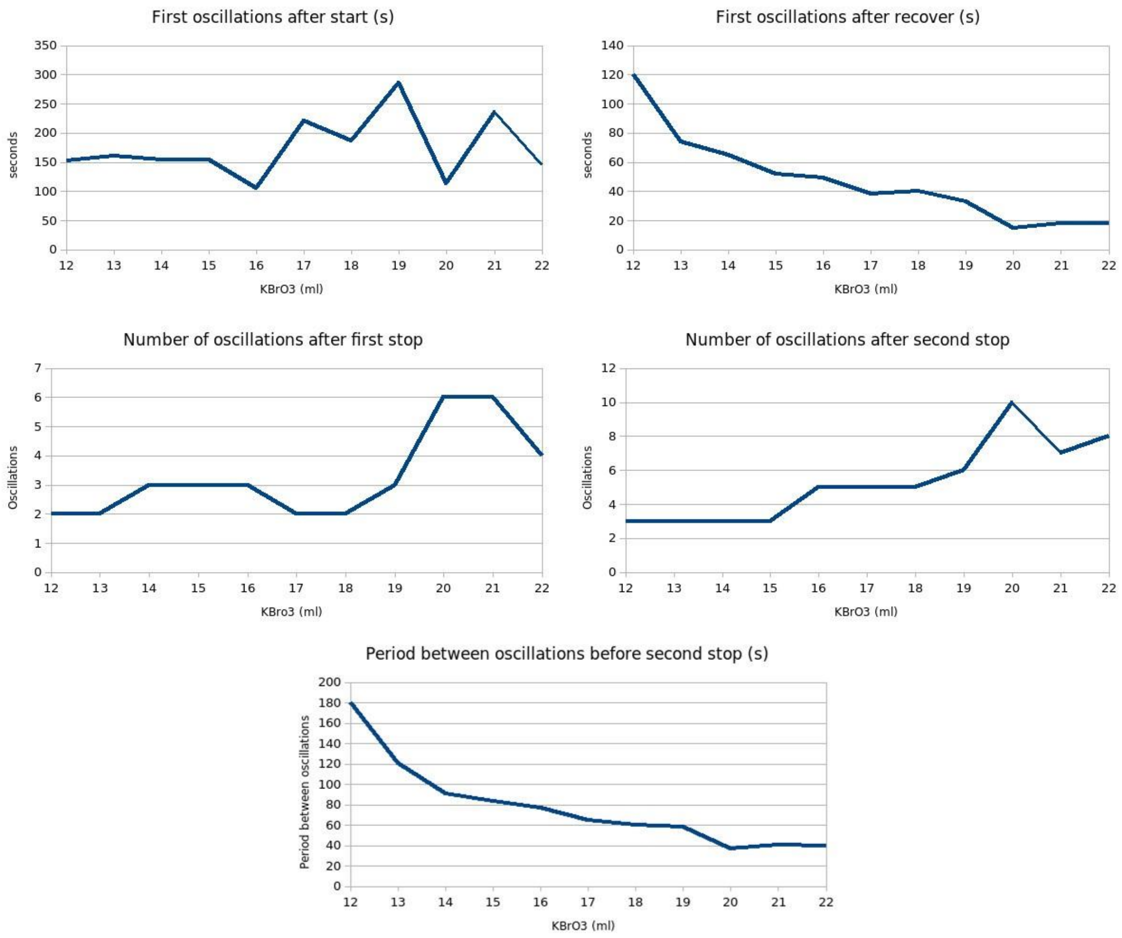

Supplementary Figure 52: Studying the number of oscillations that appear in the system once all the stirrers are disabled. Our results seem to indicate that a higher concentration of $KBrO_3$ generates a bigger “memory”.

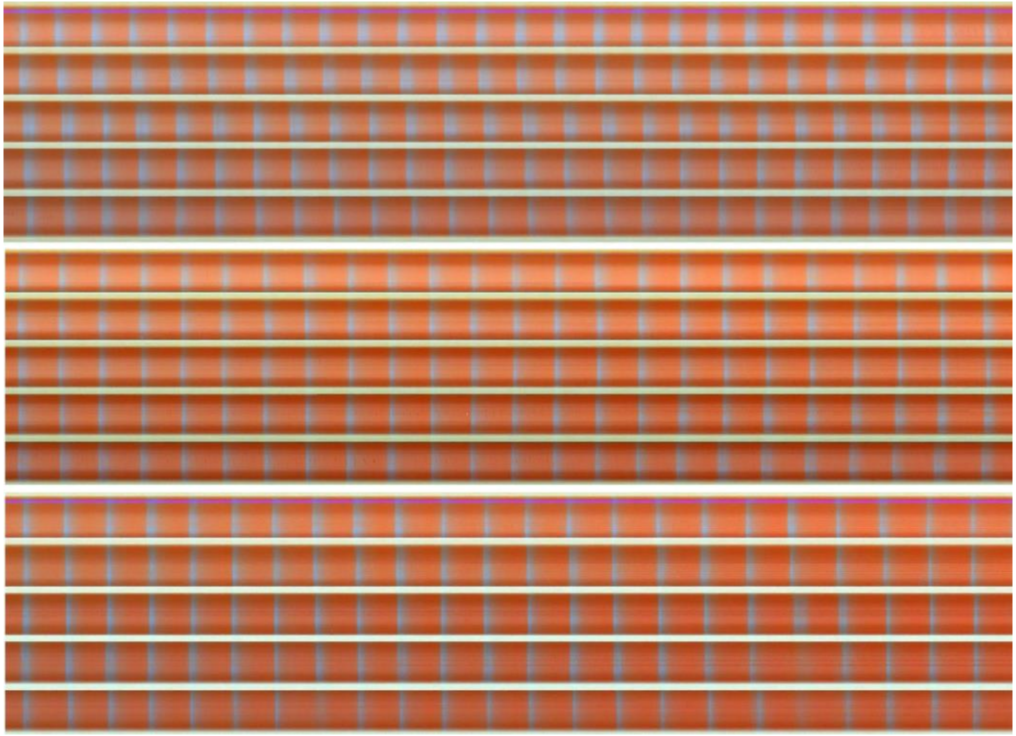

Supplementary Figure 53: Top row: default speed. Middle row: Two times default speed. Bottom row: Five times default row.

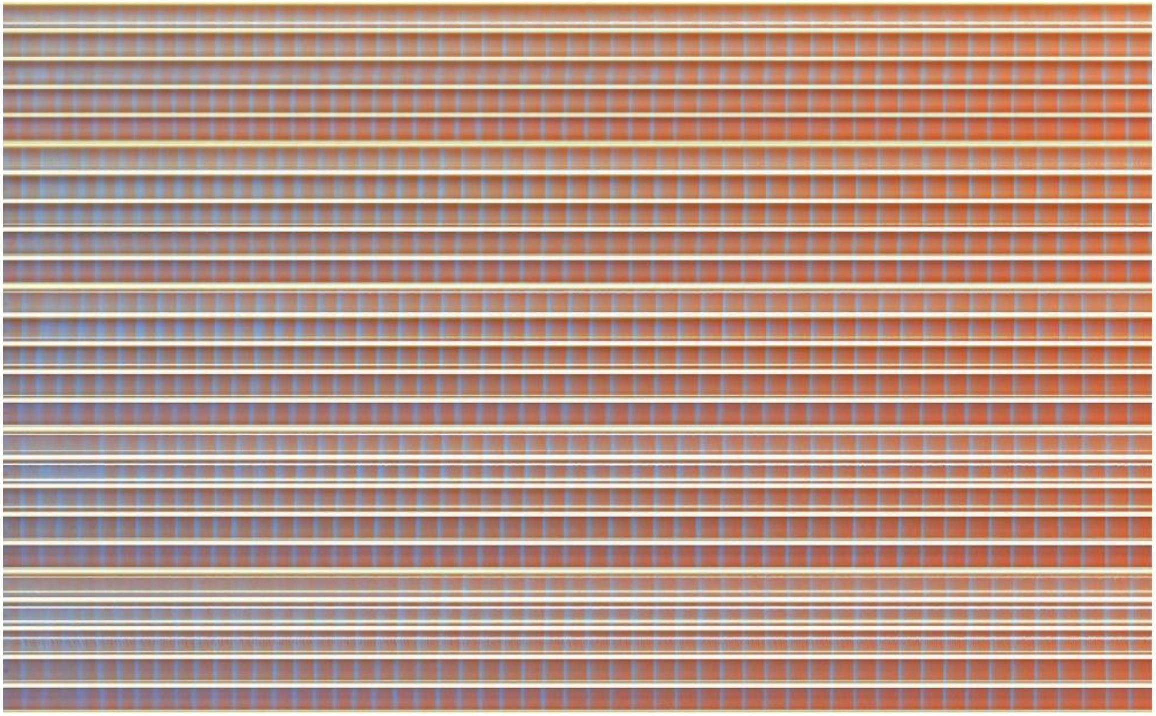

Supplementary Figure 54: In this experiment all the stirrers were set to different random speed. From completely disabled to full speed.

Setting different parts of the grid at different defined speeds

Based on these results, it was decided to test how the system would behave if within the same arena a set of stirrers was stirred at a slow speed, and a set of stirrers was stirred at a faster one, see Supplementary Figure 55. In order to do so, an experiment was designed where the first two columns rotated at the default speed (a PWM signal of 500), the third column was completely disabled, and the fourth and fifth column rotated at four times the default speed (a PWM signal of 2000).

In Supplementary Figure 55, we focused on column 2, 3 and 4. Column 2 was set a default speed (500), column 3 was completely disabled, and column 4 was at four times default speed (2000). This experiment showed that the when using this sort of pattern, the system did not arrive to a global coherent pattern, and that the part rotating at 500, and the part rotating at 2000, generated BZ oscillations at different frequencies. What was very interesting, as can be seen on this Supplementary Figure, is that only when the two systems of oscillations were in phase, then and only then would the column 3, which was disabled, oscillate. In this Supplementary Figure, this phenomena is marked with arrows.

4.8Studying the stability in the frequency of the BZ oscillations

In this section we will study how sSupplementary Table is the frequency of the BZ oscillations during a whole experiment. During this section a slightly different platform was used. This different platform contained a single row of 7 cells. Therefore, the results shown during the rest of this Section 4.8 were done with a 1 by 7 platform instead of the 5 by 5 platform used elsewhere.

Our results showed that the frequency of oscillations for each of the 7th cells was the following: {average time between oscillations in seconds, standard deviation}: {{33.0952,1.61782},{33.0397,1.05079},{33.0357,0.765763},{33.0238,0.794536},{33.0317,0.923614},{33.0119,1.13299},{33.0556,1.15451}}. Therefore, the average for the 7th cells was 33.042 seconds. Supplementary Figure 54b shows the stability of the oscillations for each cells, and as it can be seen here, it was very sSupplementary Table through all the experiment, with differences of just a few seconds between oscillations.

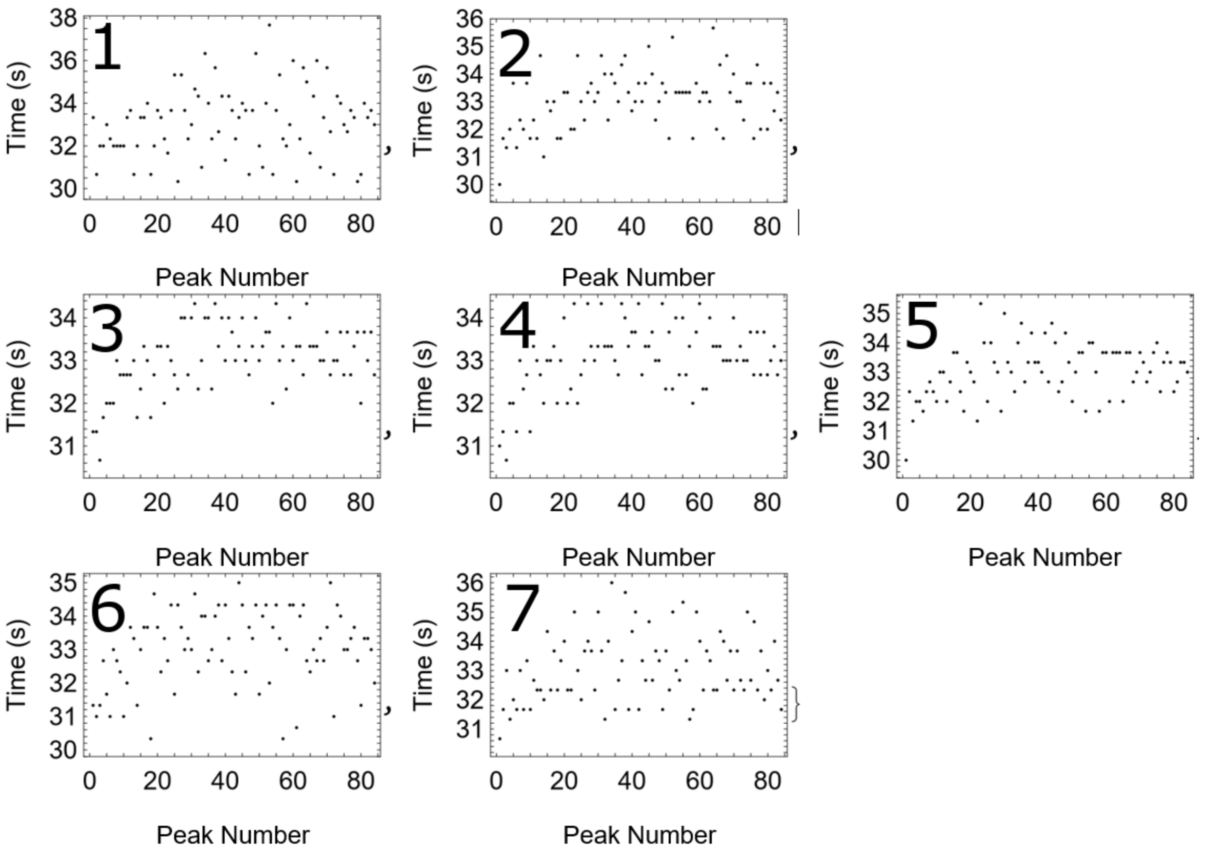

Supplementary Figure 54b: Each plot represents the time between oscillation peaks for each of the 7 cells. The columns are the peaks, while the rows is the time. As it can be seen, the frequency of oscillations was very sSupplementary Table all the way through the experiments.

4.9 Programming a pattern sequence into the BZ medium

The next objective was to study if by flashing different patterns in a sequence we could modify the global oscillation generated by the BZ medium. This idea is supported by the fact that the BZ medium has memory, as shown before (Section 4.6). Therefore, the hypothesis is, if we have a pattern A that generates a global oscillation A', and we also have a pattern B that generates a global oscillation B'; if, for example, we flash first A, and then B, we can assume that the first global oscillation generated will be A', but once it switches to B, will it be B' or something different?

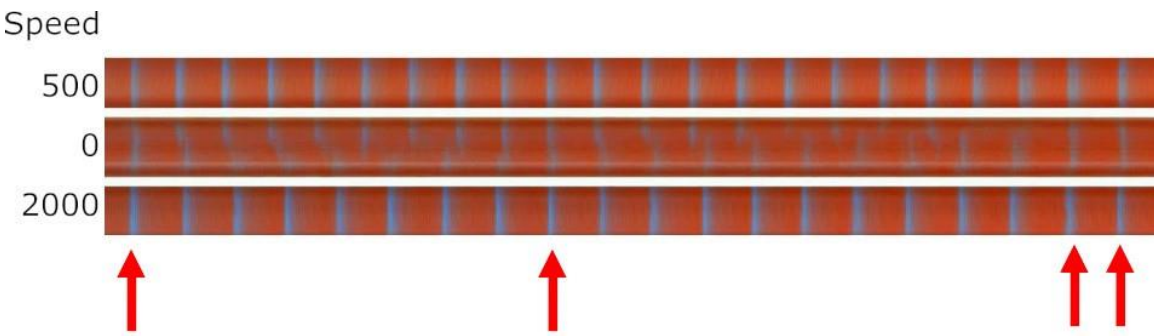

Supplementary Figure 55: Column 2 rotated at the default speed (around 80rpm, which was generated with a 500 PWM signal). Column 3 was disabled. Column 4 rotated at four times the default speed (2000 PWM). Only when the two systems were in phase would then column 3 oscillated.

To test this hypothesis, we tested it using two different experimental pipelines, shown on Supplementary Figure 56. In the first one, which is the top half sequence of this Supplementary Figure, initially we flashed a base pattern for 10 minutes, in our case, the one related to "0" (see Section 4.2). Then it followed by a period of 5 minutes were nothing was flashed. Then, there it was flashed a sequence of 3 patterns, each of them for 5 minutes. We tested for pattern 1, then pattern 2 and then pattern 3, and also for pattern 4, then pattern 5 and then pattern 6. The bottom half of this Supplementary Figure shows the other sequence of patterns we tested. In this case, patterns 0, 1, 2 and 3 looped in a 1 minute period (therefore, 30 iterations in total) where each of these patterns was flashed for 15 seconds.

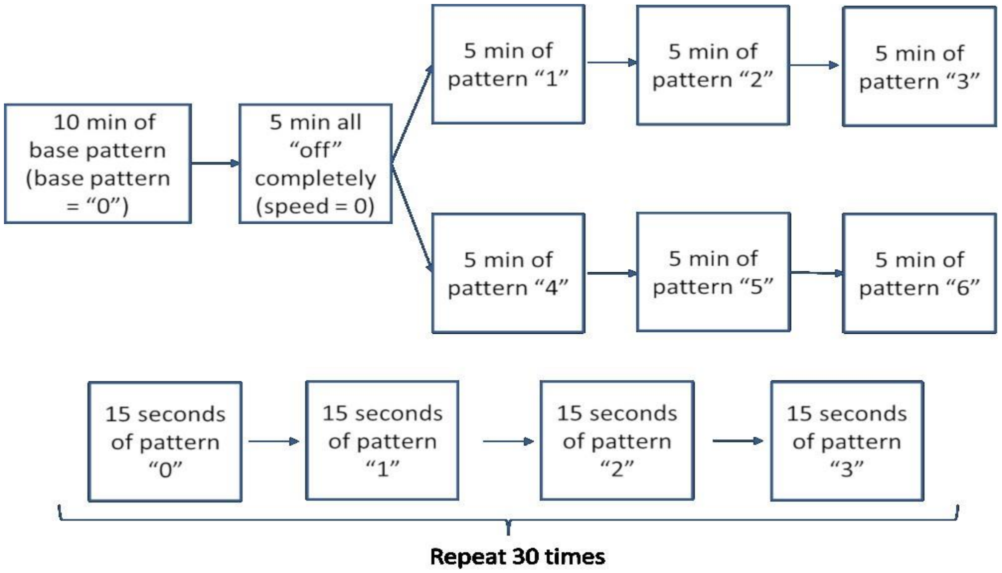

Supplementary Figure 56: In order to test the programmability of the system, we tested two different experimental pipelines. In one of them, four different patterns were flashed in a sequence, each pattern only appearing once for 5 or 10 minutes, for a total of 30 minutes. In the other one, we had 4 pattern iterating continuously in 1 minute cycles.

The results of these experimental pipelines are shown on Supplementary Figure 57. These Supplementary Figures contains a series of linear plots. In each of these plots, the lines represent the direction of the BZ global wave. For example, in the bottom left linear plot, with lines going in a diagonal from bottom left to top right, the global wave was moving in the direction suggested by these lines. In order to obtain these plots, for each frame of a experiment, we extracted the blue channel, and calculated its moment and the centre of mass. We then concatenated these centres of masses between plots, and the way the centre of mass varies between frames is what is represented in these linear plots.

In this Supplementary Figure, initially we show the waves described by the patterns 0, 1, 2 and 3 when they are the only ones flashed during 30 minutes. These plots contain red lines. Then we show the case in which these four different patterns are flashed in a continuous loop, where each pattern is flashed for 15 seconds. These plots contain green lines. Finally, we show the case where initially a pattern 0 is flashed for 10 minutes, followed by 5 minutes of nothing, and then pattern 1, then 2 and then 3, five minutes each, in a sequence. These plots contain blue lines.

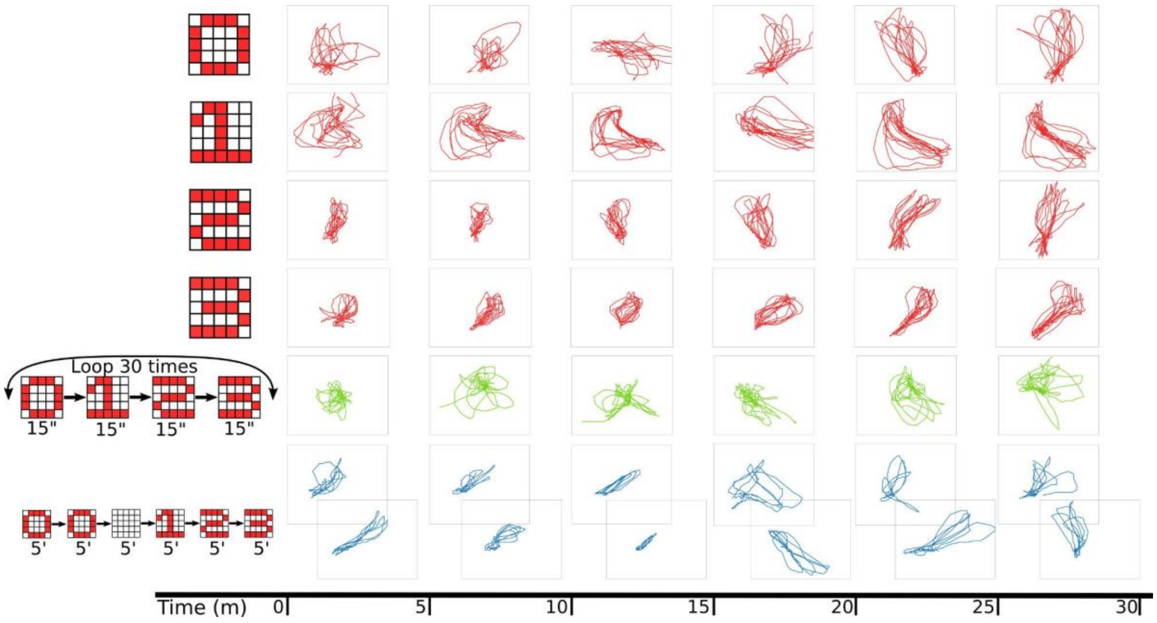

Supplementary Figure 57: Plot lines in red: patterns flashed in isolation for 30 minutes. Plot lines in green: Patterns 0, 1, 2 and 3 looped in 1 minute cycles, where every pattern was flashed for 15 seconds. Plot lines in blue: experimental pipeline where 4 different patterns were flashed in a sequence, each pattern was only flashed once for 5 or 10 minutes. While previously (red or green line plots) each plot represents 5 minutes of experiment, in this case (blue line plots) each plot represents 2.5 minutes of experiments.

In the case of the continuous loop pattern, it seems that the system never arrived to a coherent global wave. In the case of the sequence of the four different patterns, it seems that it arrived to a global wave for every pattern, and each of these global waves were different. In particular, if we compare for example the wave described by pattern 1, 2 or 3 when they are flashed in isolation, or the wave described when they were

flashed in a sequence, there it can be seen that the global waves were different. This might mean that we were able of “programming” the system by flashing a sequence of patterns, but we were never able of proving if this was actually being caused by our experimental pipeline, or if it was cause by the stochasticity of the system.

4.10 Experimenting with water medium instead of BZ medium

In order to test if the BZ medium was necessary in the system in order to perform the described computations, we executed a series of experiments where instead of BZ medium we used water. As it is shown in the main manuscript, and also described in Section 5, the platform described using BZ medium was able of encoding input patterns, such as the ones shown in Section 4.2, and then decoding them with the aid of machine learning.

In these experiments with water, the objective was to see if the water medium was also able of encoding input patterns, that later on were decoded with the aid of machine learning. In this case, the patterns for “0” and “4” were flashed into the system by stirring those motors with the defined pattern. Then a 30 minute video was recorded of the arena (see Supplementary Figure 58 A). For each of the 25 cells, we extracted its RGB value through time, and transformed it into the HSL colour scheme. Supplementary Figure 58 B shows the brightness level of the 25 cells during the experiment. The scale goes from 0 to 255, but here we are focusing on values between 150 and 185. In this Supplementary Figure, there it can be seen that the brightness variation through the experiment is minimal because the system was not oscillating. With this data, we input it to our machine learning model, in order to decode it, but our test performance was around 50%, therefore the decoding phase was completely random. These results then show that a chemical oscillating system is necessary in order to compute.

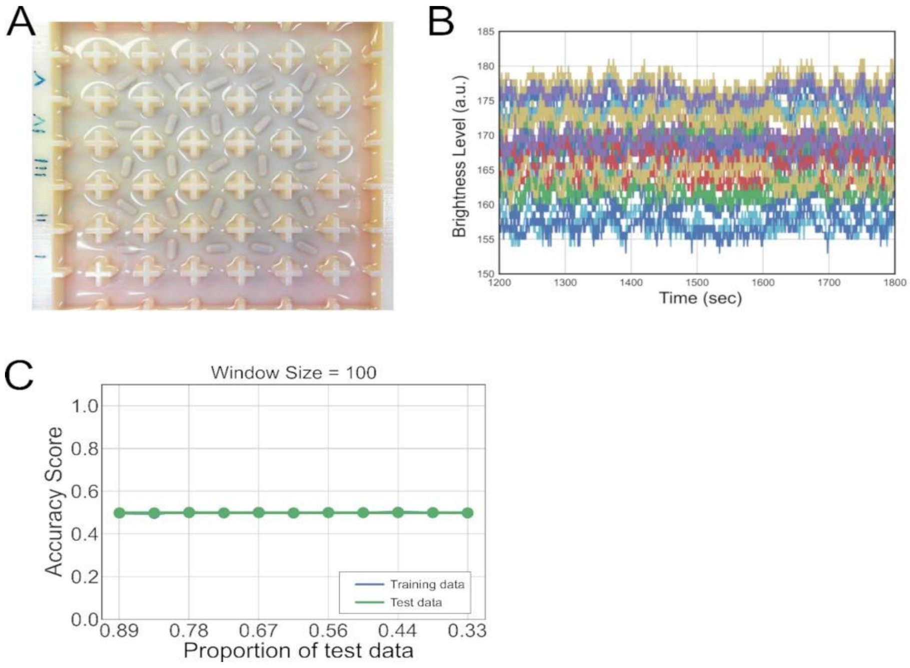

Supplementary Figure 58: Water experiment. (A) A raw camera image of the experiment. Pure water was added in the grid instead of BZ reaction medium. (B) Example time-series data from the experiment. (C) Results of prediction for two input patterns (“0” and “4” as shown in Section 4.2). Note that the training data and test data were overlapped each other, thus only the test data (green line) was visible.

5 Data analysis

Supplementary Source Code 7: CNN specification

```
height = 25
width = 25
channels = 1
n_inputs = height * width

conv1_fmaps = 2
conv1_ksize = 3
conv1_stride = 1
conv1_pad = "SAME"

conv2_fmaps = 2
conv2_ksize = 5
conv2_stride = 1
conv2_pad = "SAME"
conv2_dropout_rate = 0.5

conv3_fmaps = 1
conv3_ksize = 3
conv3_stride = 1
conv3_pad = "SAME"
conv3_dropout_rate = 0.5

n_fc1 = 16
fc1_dropout_rate = 0.5

n_outputs = 10
```

5.3 Using CNN to train the BZ medium

In order to extract the rules of the computation of the BZ medium, a Convolutional Neural Network (CNN) was used. The CNN implementation from Tensorflow was used. The scripts used are based on the book "Hands-on machine learning with Scikit-Learn and Tensorflow"[1]. In this case, 20 patterns different representing numbers, letters and random configurations were used (see Section 4.2). In this case, CSV used were the binarised ones. Therefore, each of these CSV had values 0 or 1. These CSV contain 3600 values. In this case, values from 1000 to 3600 were used. The window size used was 30. Therefore, the inputs to the CNN were 25 by 30, or 750 values. The test and train ratio were set to 30%. This CNN contained 3 convolutional layers with 32 filters of size 5, 64 of size 3, and 128 of size 3. Finally, it had a fully connected layer with 64 neurons. Finally, there is a fully connected layer with 64 neurons, and then 20 output neurons. See Supplementary Source Code 7 and 8 for examples of CNN specifications, the actual values used are like the ones described here in the text, and now the ones shown in those Supplementary Source Codes. The CNN was trained and executed following the code snippets shown in the CNN chapter of "Hands-on machine learning with Scikit-Learn and Tensorflow"[1]. Once the model was trained, the last step was to extract the feature maps the kernels from each of the layers. In order to increase the accuracy to 92.5%, a train/test split of 10% was used, using a moving window size of size 20 (frames) by 25 cells (this 500 features). The architecture of this network was at follows: Conv1 16 feature maps, kernel of size 5, dropout 50%. Conv2 identical but no dropout. Conv3 identical with dropout 50%. Maxpool layer with kernel of size 2 by 2 and strides of size 2 by 2. Conv4 32 feature maps and kernel of size 5, dropout 50%. Conv5 identical but no dropout. Conv6 identical with dropout 50%. Maxpool layer with kernel of size 2 by 2 and strides of size 2 by 2. Conv7 64 feature maps and kernel of size 3, dropout 50%. Fully connected layer with 64 neurons. Softmax layer.

5.4 Using an Autoencoder to digitally generate BZ oscillations

In order to create a neural network that could digitally generate BZ oscillations, we used a model similar to the Autoenconder, where the input of the network the motor array patterns (see Section 4.2) and the output of this network were BZ oscillations as generated from the medium. Therefore, this network will learn to given a motor configuration, generate a BZ oscillation.

The Tensorflow library was used. The scripts used are based on the book "Hands-on machine learning with Scikit-Learn and Tensorflow"[1]. In this case, in order to simplify the system, the patterns representing numbers from 0 to 9 were used (see Section 4.2) In this case, CSV used were the binarised ones. Therefore, each of these CSV had values 0 or 1. These CSV contain 3600 values.

The Autoenconder used had 25 inputs and 625 outputs. Three hidden layers were used of 50, 10 and 50 neurons. The code used to train this autoencoder was "Training One Autoenconder at a Time" from [1]. Each autoenconder was trained for 3000 iterations. The learning rate was 0.00001 and L2 regularization was used (0.00001). The output from this autoenconder was input into the CNN explained in Section 5.3.

Supplementary Source Code 8: CNN layers declarations

```
with tf.name_scope("inputs"):
  X = tf.placeholder(tf.float32, shape=[None, height, width], name="X")
  X_reshaped = tf.reshape(X, shape=[-1, height, width, channels])
  y = tf.placeholder(tf.int32, shape=[None], name="y")
  training = tf.placeholder_with_default(False, shape=[], name='training')

conv1 = tf.layers.conv2d(X_reshaped, filters=conv1_fmaps, kernel_size=conv1_ksize,
↪  strides=conv1_stride, padding=conv1_pad, activation=tf.nn.relu, name="conv1")
conv2 = tf.layers.conv2d(conv1, filters=conv2_fmaps, kernel_size=conv2_ksize, strides=conv2_stride,
↪  padding=conv2_pad, activation=tf.nn.relu, name="conv2")
conv3 = tf.layers.conv2d(conv2, filters=conv3_fmaps, kernel_size=conv3_ksize, strides=conv3_stride,
↪  padding=conv3_pad, activation=tf.nn.relu, name="conv3")

with tf.name_scope("pool3"):
  pool3_flat = tf.reshape(conv3, shape=[-1, pool3_fmaps * heigth * width])
  pool3_flat_drop = tf.layers.dropout(pool3_flat, conv2_dropout_rate, training=training)

with tf.name_scope("fc1"):
  fc1 = tf.layers.dense(pool3_flat_drop, n_fc1, activation=tf.nn.relu, name="fc1")
  fc1_drop = tf.layers.dropout(fc1, fc1_dropout_rate, training=training)

with tf.name_scope("output"):
  logits = tf.layers.dense(fc1, n_outputs, name="output")
  Y_proba = tf.nn.softmax(logits, name="Y_proba")
```

5.5 Mutual information calculations

The mutual information calculations shown on Supplementary Table 3 were performed using the function "normalized mutual info score" from "sklearn.metrics.cluster".

	pat0	pat1	pat2	pat3	pat4	pat5	pat6	pat7	pat8	pat9
pat0		0.005	0.058	0.058	0.001	0.064	0.281	0.036	0.281	0.028
pat1	0.120		0.005	0.005	0.053	0	0.005	0.442	0.005	0.005
pat2	0.031	0.279		0.365	0.001	0.096	0.204	0.116	0.365	0.058
pat3	0.093	0.280	0.401		0.001	0.383	0.204	0.116	0.365	0.058
pat4	0.258	0.133	0.032	0.056		0.039	0.031	0	0.001	0.009
pat5	0.008	0.165	0.107	0.133	0.043		0.383	0.058	0.211	0.064
pat6	0.0375	0.219	0.125	0.081	0.090	0.234		0.036	0.597	0.058
pat7	0.101	0.376	0.199	0.161	0.183	0.219	0.326		0.0365	0.009
pat8	0.199	0.229	0.174	0.250	0.167	0.093	0.178	0.229		0.146
pat9	0.075	0.149	0.227	0.216	0.057	0.046	0.054	0.136	0.226	

Supplementary Table 3: Top-right part of the Supplementary Table, mutual information of the motor patterns as described in Section 4.2. Bottom-left part of the Supplementary Table, mutual information of the BZ oscillations produced by said patterns, after being trained in an Autoenconder. Therefore, the data here being compared is the "outputs" from Supplementary Figure 5B in the main manuscript.

6 Towards generalized computation using the BZ platform

The experimental platform consists of n x n cells in a square grid with nearest neighbours connected to each other with an interconnecting channel of the defined dimensions. Each cell consists of a magnetic stirrer together with the motor connected to PWM input. The oscillation amplitude and frequency of Belousov Zhabotinsky (BZ) reaction usually depend on the stirring rate and temperature. In this section, based on the certain assumptions, we explore the possible experimental observables in our experimental set-up which can be utilized for computational operations such as pattern recognition, neural networks etc.

6.1 Experimental inputs

The inputs of the BZ computational platform are defined by the vector with cell positions and the stirring rate of the stirrer placed in the cell. Activating a stirrer in a cell not only affects the amplitude and oscillating

frequency of BZ reaction but also allows interaction between nearest neighbours, which can be seen as an operation for information transfer and processing. Assuming each cell (C_{ij}) can be activated with stirring action with k different PWM levels capable of creating distinct chemical oscillations. The total number of input possible combinations are given by k^{nxn}. For the case of two different levels (ON and OFF), the minimum number of combinations are 2^{nxn}. The scaling of the number of input states with n (number of cells in a row/column) is shown in the Supplementary Figure 59A.

6.2 Chemical states

We define the chemical state of the BZ computational platform using an oscillatory amplitude of each cell which can be utilized as a basic chemical information processing unit. The single chemical state of the system can be defined for a defined subset of the computational cell by discretizing the amplitude in regular intervals between RED to BLUE states.

If we assume for simplicity, that each cell can oscillate between *p* different levels above base RED state, the maximum number of chemical states is given by $(1+p)^{nxn}$ where additional state comes when there are no oscillations. Formation of synchronized global waves or independent oscillating regions are different combinations of phase differences and amplitudes of BZ cells. The scaling of the number of chemical states with *n* (number of cells in a row/column) is shown in the Supplementary Figure 59B. Supplementary Table 4 shows estimated states and other parameters for BZ computational platform.

Parameter	Magnitude	Comments
**Input States (MIN)**	*ca.* 3.3×10^{7}	Assuming two different voltage levels (ON/OFF) for the stirrers (no PWM) on 5 x 5 grid.
**Input States (MAX)**	*ca.* 1.1×10^{15}	Assuming 4 different PWM levels on 5 x 5 grid, with each level capable of modulating the amplitude and phases of BZ oscillations locally in the cell.
**Chemical States (MAX)**	*ca. 2*$.9\times10^{17}$	Assuming four different oscillatory amplitudes between red and blue states at a given time on 5 x 5 grid processing cells. The chemical processing states can also be defined using considering different frequencies of oscillations as well. [2, 3, 4, 5]
**Chemical Memory**	25 bits	Assuming 5 x 5 grid with one bit of information on each cell
**Chemical Information readout rate**	2.5 bits/s	Assuming the fastest oscillation frequency is 1/10 Hz with imaging full 5 x 5 grid.

Supplementary Table 4: Relation between the input and the chemical states.

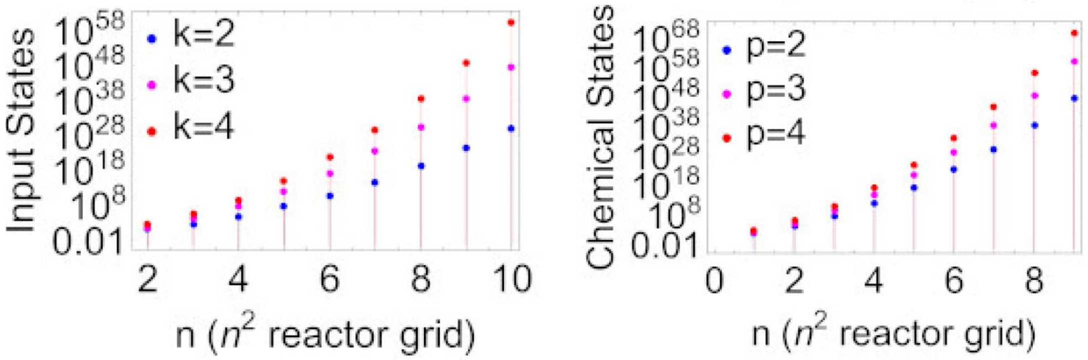

Supplementary Figure 59: Scaling of the number of states in the BZ computational platform. (A) shows the scaling of the number of input states with the number of BZ cells on the experimental platform at different PWM stirring inputs (2,3 and 4). (B) shows scaling of the number of chemical states with the number of BZ cells on the experimental platform at a different number of measurable oscillatory amplitudes (2, 3 and 4).

6.3 Mathematical description of the dynamics of coupled BZ Oscillators

In this section, we discuss the coupling between various physicochemical effects expected in the experimental platform and describe a phenomenological model which can be used to develop the efficient and optimized design of the platform. BZ oscillations are often modelled using the mathematical description of autocatalytic reactions such as Brusselator or Oregonator. Due to the complexities of stirring effects which includes the effect of stirring rate on amplitude and frequency of BZ reaction as well as mass transfer between neighbouring cells, we use phase dynamics scheme often used for weakly coupled oscillators. Here, we define set of assumptions to simplify the complexity of coupling between chemical reactions with hydrodynamic interactions and define computation relevant operations on BZ grid.

1. **BZ Cell description:** The time-dependent state of each BZ cell is defined by the phase and amplitude of the oscillation. In the absence of an interaction between neighbouring cells, each cell oscillates at its natural frequency defined by the stirring rate which is also a time-dependent operation. So, the complete experimental platform at any time is defined by stirring rate of each cell which defines the natural frequency and phase information. We assume only two different measurable states (RED/BLUE) and hence neglect the amplitude modulation, see Supplementary Table 4.

2. **Defining Cell States: ACTIVE, PROCESS and INACTIVE** Similar to the experiments and to define different field-programmable computational operations, we categorized the cell into three different states, active, process and inactive.

- **ACTIVE** cells have relatively faster stirring speed which initiates strong oscillations and influences the neighbouring cells. These cells act as inputs for information processing in the BZ grid.
- **PROCESS** cells have lower stirring speed which is not capable of initiating strong oscillations but allows and enhances interaction between neighbouring cells. These cells transfer information and process information from the ACTIVE and other PROCESS cells.
- **INACTIVE** cells have stirrers turn off which inhibits the information transfer and with the neighbouring cells. INACTIVE cells help in allowing parallel information processing in different regions on the grid.

3. **Cell-Cell Interactions:** The interaction between the neighbouring cells depends on the cell state as described above (2). Due to the different stirrer rates in ACTIVE, PROCESS and INACTIVE cells, there is asymmetric mass transfer between the neighbouring cells. We assume cell-cell interactions are confined to nearest-neighbours only, such that ACTIVE and PROCESSING cells can only influence the four nearest neighbours in a square grid.

4. **Coupling Constants between different cell types:** The coupling constant quantifies the cell-cell interaction between two cells neighbouring cells, which can be used as an interaction parameter in the mathematical description of phase evolution. The coupling constant ($K_{ij\rightarrow mn}$) between the two neighbouring cells (indicated by (i,j) and (m,n)) depends on the cell types as described above (2). As different cell types have different speed of the stirrers, there in an asymmetric information transfer between the neighbouring cells. So, depending on the different possible combinations of neighbouring cell types, we can define coupling constants which can be used as parameters which depends on the physical design and angular velocity of the stirrers.

$$K_{ij\rightarrow mn} = \begin{cases} K_1, & ij = ACTIVE \text{ and } mn = ACTIVE \\ K_2, & ij = ACTIVE \text{ and } mn = PROCESS \\ 0, & ij = ACTIVE \text{ and } mn = INACTIVE \\ \\ 0, & ij = PROCESS \text{ and } mn = ACTIVE \\ K_3, & ij = PROCESS \text{ and } mn = PROCESS \\ 0, & ij = PROCESS \text{ and } mn = INACTIVE \\ \\ 0, & ij = INACTIVE \text{ and } mn = ACTIVE \\ 0, & ij = INACTIVE \text{ and } mn = PROCESS \\ 0, & ij = INACTIVE \text{ and } mn = INACTIVE \end{cases}$$

So, in the current model, there are only three different interactions, between two ACTIVE cells, from ACTIVE to PROCESS cells and between two PROCESS cells. Using $K_1(r_s)$, $K_2(r_s)$ and $K_3(r_s)$, we can construct a network of coupled oscillators and investigate the evolution of different chemical states.

5. **Memory effect:** As observed in experiments, once a pattern is created on the BZ grid, on deactivating the INPUT cells, the global periodic oscillations sustains for certain time which eventually dampens.

The presence of this memory effect in oscillations could be utilized to apply time-dependent operations on the previous state which could be utilized to build a set of complex operations.

Based on the above description, each BZ cell of the grid is defined by an ordinary differential equation (ODE) for phase dynamics with coupling with nearest neighbours[6]. We consider a two-dimensional array of coupled oscillators in a square lattice, so the evolution of phase of BZ cell $\varphi(i,j)$ is given by,

$$\frac{d\phi_{ij}}{dt} = \sum_{mn} H\left(\phi_{mn} - \phi_{ij}\right) K_{ij\to mn}\left(r_s\right) + \omega_{ij} + \xi(t)$$

where, φ_{ij} is the phase of cell C_{ij}, ω_{ij} is the natural oscillation frequency which is a function of the angular velocity of the stirring, $K_{ij\to mn}$ (r_s) is the coupling constant between the two neighbouring cells which also depends on the angular velocity of the stirrer (r_s) and the physical design of the interconnects between the two cells. The stronger the angular velocity of the stirrer, stronger the fluid flow and mass transfer between two interconnected cells, and hence larger the coupling constant. $\xi(t)$ is the common noise which satisfies $<\xi(t)\xi(t')> = 2D\delta(t - t')$.

These set of coupled equations are complicated by the hydrodynamics between the cells and non-linearity of the Belousov-Zhabotinsky reaction. For simplicity, we assume H as a sinusoidal or a continuous rectangular wave function with a defined period, the above set of equations can be solved using an explicit finite difference scheme. Using these calculations, we can investigate the evolution of different chemical states together with operations and control over the BZ grid. See Supplementary Figure 60.

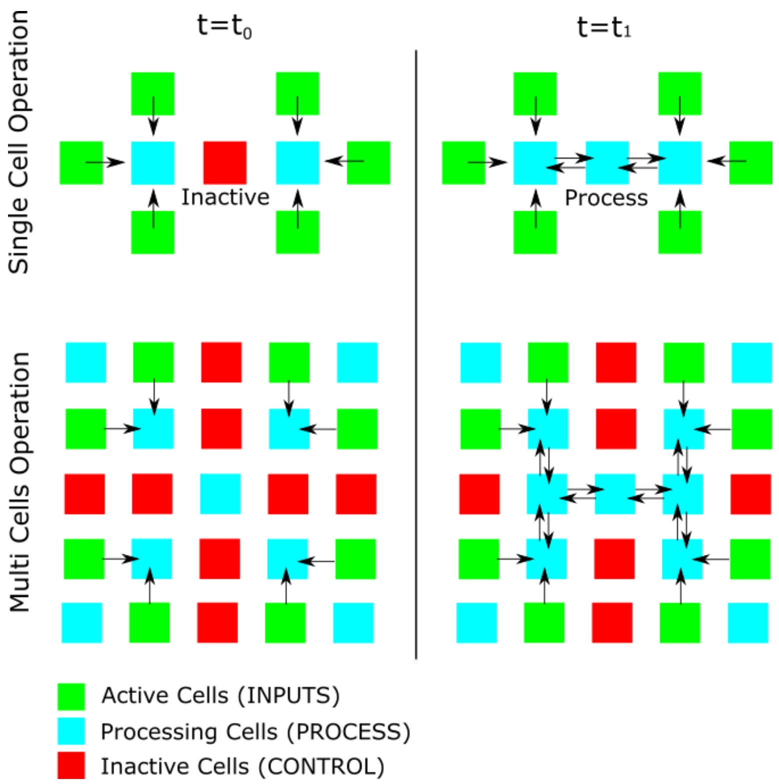

Supplementary Figure 60: Operations on BZ grid. Supplementary Figure shows time-dependent single cell or multi-cell operations on the BZ grid by tuning the stirring rates of individual cells.

7 Estimating number of operations and required logic gates

BZ Platform (5 by 5 array):

(Assumption: Two possible initial phases (Red/Blue) and four different oscillation frequencies)

- Total Number of Input Patterns (Binary Stirring Patterns): $2^{25} = 3.35 \times 10^7$
- Total Number of Chemical States (defined by Initial Phase and Oscillation Frequency): $(4+1)^{25} = 2.9 \times 10^{17}$

Autoencoder: Three layers (50-10-50 Neurons):

- Estimated Gate Count for fast multiplication: 12928 [7]
- Estimated Gate Count for addition operation: 1440 [8]
- Possible Input Patterns to autoencoder: $2^{25} = 3.35x10^7$
- Possible Output Patterns from autoencoder: 2^{625} (25 cells x 25 time slices centred around oscillation peaks)

Layer 1:

- Number of Neurons: 50
- Number of Inputs: 25
- Total Multiplication Operations: 50 x 25 = 1250
- Estimated Total Number of Gates for Multiplication Operations: 1250 x 12928 = 1.616×10^{7}
- Total Addition Operations: 50 x 25 = 1250
- Estimated Total Number of Gates for Addition Operations: 1250 x 1440 = 1.8×10^{6}
- Total Number of Activation Operations: 50

Layer 2:

- Number of Neurons: 10
- Number of Inputs: 50
- Total Multiplication Operations: 10 x 50 = 500
- Estimated Total Number of Gates for Multiplication Operations: 500 x 12928 = 6.464×10^{6}
- Total Addition Operations: 10 x 50 = 500
- Estimated Total Number of Gates for Addition Operations: 500 x 1440 = 7.2×10^{5}
- Total Number of Activation Operations: 10

Layer 3:

- Number of Neurons: 50
- Number of Inputs: 10
- Total Multiplication Operations: 50 x 10 = 500
- Estimated Total Number of Gates for Multiplication Operations: 500 x 12928 = 6.464×10^{6}
- Total Addition Operations: 50 x 10 = 500
- Estimated Total Number of Gates for Addition Operations: 500 x 1440 = 7.2×10^{5}
- Total Number of Activation Operations: 50

Totals:

- Total Number of Neurons: 50 + 10 + 50 = 110

- Total Multiplication Operations: 1250 + 500 + 500 = 2250
- Total Addition Operations: 1250 + 500 + 500 = 2250
- Total Number of Activation Operations: 50 + 10 + 50 = 110
- Total Operations: 110 + 2250 + 2250 + 110 = 4610
- Estimated Total Number of Gates for Multiplication Operations: 2.9088 x 10^7
- Estimated Total Number of Gates for Addition Operations: 3.24 x 10^6
- Estimated Total Number of Gates: 3.23 x 10^7 (32.3 Mi Logic Gates)
- Assuming the minimum of 2 transistors per logic gate (3 for AND and OR, 2 for NAND and NOR), the total number of equivalent transistors are 32.3 Mi x 3 = 64.6 Mi or ca. 60 Mi
- Assuming the maximum of 3 transistors per logic gate (3 for AND and OR, 2 for NAND and NOR), the total number of equivalent transistors are 32.3 Mi x 3 = 96.9 Mi or ca. 100 Mi
- So, at the individual transistor scale there are a minimum of 60 Mi operations possible or a maximum of 100 Mi operations possible.
- Assuming that our BZ platform encodes similarly to the described Neural Network using 1 minute window of data, then it encodes an equivalent of 60 M/60s = $1Ms^-1$.

8 Power dissipation in BZ reaction

$$3CH_2(COOH)_2 + 4BrO_3^- \rightarrow 4Br^- + 9CO_2 + 6H_2O$$

Enthalpy of formation (from NIST database):

1. Malonic Acid (MA): -634.87 kJ/mol
2. Bromate: -342.50 kJ/mol
3. Bromide: -121.0 kJ/mol
4. Carbon dioxide: -393.50 kJ/mol
5. Water: -285.82 kJ/μmol

Using Hess' Law:

- For Reactants: 3 MA + 4 BrO_3^- = -3274.61 kJ/mol

- For Products: 4 Br^- + 9CO_2 + 6H_2O = -5619.42 kJ/mol
- Difference = -2344.81 kJ/mol (for 3 mols of Malonic Acid)
- For per mol of malonic acid, enthalpy of reaction is -781.60 kJ/mol

Concentration of Malonic Acid: 1 M
Total volume of Malonic Acid used: 18ml Total time: ca.
2.5 hours **(Assumed)**

So, the total power dissipation is given by,
P = (781.60 kJ/mol x 1 M x 18 ml) / (2.5 x 3600s) = 1.56 J/s

If we use the BZ platform to run 50 x 10 x 50 neural network with the estimated speed of 1 Mi equivalent transistor operations per second, the power consumption per operation is 1.56 μJ. However, running a three-layer 50 x 10 x 50 neural network only uses a small subset of total possible chemical states (as most of the BZ cells oscillates at the same frequency with variable phase differences). So, with the similar power consumption, much more information processing is possible. So, accessing all chemical states (2 phase states, 4 frequencies give 3.77 x 10^{22}) can substantially reduce the power consumption per chemical operation. It is important to note that, by correctly defining a chemical operation, much more information processing is possible as compared to single transistor scale or logic operations.

If we assume that all chemical states are accessible in 2.5 hours, the estimated power consumption per chemical operation (switching form one chemical state to other),
P = (781.60 kJ/mol x 1 M x 18 ml) / (2.5 x 3600s) x ($1/2^{25}4^{25}$) = 4.13 x 10^{-23} J/s

Current state-of-the-art, Intel i9 processor with 7 Bn transistors all clocking at 4 GHz consumes 147 W. So, power consumption per operation is given by,
P = 147 W / (4 GHz x 7 x 10^9) = 5.25 x 10^{-18} J

Supplementary Note 1: Supplementary Figure enhancements

All the Supplementary Figures shown in the main manuscript and in this document were designed using Inkscape
(inkscape.org).

On Supplementary Figure 2 from the main manuscript, the top-down pictures of the device were the oscillations can be seen were manually enhanced in order to make the oscillations more visible. In the Supplementary Video these oscillations can be clearly seen.

Supplementary References

[1] Geron, A.: Hands-on machine learning with Scikit-Learn and Tensorflow, *O'Reilly* (2017).

[2] Kalishyn, Y.Y., and Rachwalska, M., Strizhak, P.E.: Stirring Effect on the Belousov-Zhabotinsy Oscillating Chemical Reactions in a Batch. Experimental and Modelling, *A Journal of Physical Sciences*, 65a, 132–140 (2010).

[3] Ruoff, P.: Excitations induced by fluctuations: an explanation of stirring effects and chaos in closed anaerobic classical Belousov-Zhabotinskii systems, *The Journal of Physical Chemistry*, 97(24), 6405–6411 (1993).

[4] Dutt, A.K., Mueller, S.C.: Effect of stirring and temperature on the Belousov-Zhabotinskii reaction in a CSTR, *The Journal of Physical Chemistry*, 97(39), 10059–10063 (1993).

[5] Noszticzius, Z., Bodnar, Z., Garamszegi, L., Wittmann, M.: Hydrodynamic turbulence and diffusion-controlled reactions: simulation of the effect of stirring on the oscillating Belousov-Zhabotinskii reaction with the Radicalator model, *The Journal of Physical Chemistry*, 95(17), 6575–6580 (1991).

[6] Hoppensteadt, E.M., Izhikevich, E. M.: Weakly Connected Neural Networks, *Springer, New York* (2007).

[7] Jones, D.W.: CS:2630, Computer Organization Notes. Beyond Integer Addition and Subtraction, *The University of Iowa - Department of Computer Science*.

[8] Jones, D.W.: CS:2630, Computer Organization Notes. Arithmetic and Logic, *The University of Iowa - Department of Computer Science*.
