# Supplementary material for: A programmable chemical computer with memory and pattern recognition
Source: Nat Commun. 2020 Mar 18;11:1442. doi: 10.1038/s41467-020-15190-3 (PMC7080730; doi:10.1038/s41467-020-15190-3)
Supplement: Supplementary file 3 — Description of Additional Supplementary Files [file 41467_2020_15190_MOESM3_ESM.docx]

File Name: Supplementary Video 1

Description: This video shows a trailer of the working platform where the different steps to perform an experiment can be seen.

File Name: Supplementary Video 2

Description: This video shows how the memory effect of the BZ reaction works.

File Name: Supplementary Video 3

Description: This video shows how the platform can be used as a computer screen when the cells are disconnected between them and therefore the oscillations do not propagate between cells.

File Name: Supplementary Video 4

Description: This is a conceptual video showing how the encoding and decoding of patterns works in our platform.
